# A Comparative Review of Natural and Synthetic Biopolymer Composite Scaffolds

**DOI:** 10.3390/polym13071105

**Published:** 2021-03-30

**Authors:** M. Sai Bhargava Reddy, Deepalekshmi Ponnamma, Rajan Choudhary, Kishor Kumar Sadasivuni

**Affiliations:** 1 Center for Nanoscience and Technology, Institute of Science and Technology, Jawaharlal Nehru Technological University, Hyderabad 500085, India; msbhagi96@gmail.com; 2 Center for Advanced Materials, Qatar University, Doha P.O. Box 2713, Qatar; deepalekshmi@qu.edu.qa; 3 Rudolfs Cimdins Riga Biomaterials Innovations and Development Centre of RTU, Faculty of Materials Science and Applied Chemistry, Institute of General Chemical Engineering, Riga Technical University, Pulka St 3, LV-1007 Riga, Latvia; rajandeshwal@gmail.com; 4 Baltic Biomaterials Centre of Excellence, Headquarters at Riga Technical University, LV-1007 Riga, Latvia; 5 Center for Composite Materials, National University of Science and Technology “MISiS”, 119049 Moscow, Russia

**Keywords:** scaffolds, tissue engineering, natural biopolymer, synthetic biopolymer, biodegradability

## Abstract

Tissue engineering (TE) and regenerative medicine integrate information and technology from various fields to restore/replace tissues and damaged organs for medical treatments. To achieve this, scaffolds act as delivery vectors or as cellular systems for drugs and cells; thereby, cellular material is able to colonize host cells sufficiently to meet up the requirements of regeneration and repair. This process is multi-stage and requires the development of various components to create the desired neo-tissue or organ. In several current TE strategies, biomaterials are essential components. While several polymers are established for their use as biomaterials, careful consideration of the cellular environment and interactions needed is required in selecting a polymer for a given application. Depending on this, scaffold materials can be of natural or synthetic origin, degradable or nondegradable. In this review, an overview of various natural and synthetic polymers and their possible composite scaffolds with their physicochemical properties including biocompatibility, biodegradability, morphology, mechanical strength, pore size, and porosity are discussed. The scaffolds fabrication techniques and a few commercially available biopolymers are also tabulated.

## 1. Introduction

Tissue engineering (TE) is the in vitro construction of bioartificial tissues and in vivo modification of cell growth and function through the implantation of appropriate cells isolated from donor tissues to generate biocompatible scaffold materials [1]. This approach specifically focuses on the vital imbalance between the rising number of patients waiting for organ transplantation due to end-stage failure and a limited number of donated organs available for those procedures [2]. TE and regenerative medicine integrate information and technology from various fields such as genetics, engineering, pharmaceutics, medicine, chemistry, and materials sciences to perform treatments or to restore or replace damaged tissues and organs [3,4,5]. It holds the promise of sustainable development due to ever-going improvement in biomaterials and implies the procedure of fusing scaffolds, molecules, and cells that are biologically active into functional tissues. The ultimate goal is to completely monitor, create a functional structure/support to repair, preserve, or improve damaged tissues or entire organs and to implement “enhanced and sustainable quality of life (QOL) with health” as stated in the prime goal of the World Health Organization (WHO) [6,7].

In this field, two primary approaches are used to generate engineered tissues. Primarily, scaffolding is used as a cell supporting system for seeding cells in vitro, and further cells are stimulated to set up the matrix for building a tissue base for transplantation. The latter entails the use of a scaffold as a drug delivery device or a growth factor. This approach combines scaffolding with growth factors, and the body implant cells are recruited around the matrices at the scaffold sites to form the neo-tissue. Both methods do not preclude one another and can be easily fused [8,9,10,11].

Owing to its remarkable merits, TE is often believed to be the ultimate ideal medical treatment. This process is multi-stage and requires the development of various components to create the desired neo-tissues or organs. In several current strategies, biomaterials are essential components. The recent development of TE involves the preparation of new biomaterials that can meet the local environment and indications. Advanced technologies are now available to fabricate biomaterials (natural/synthetic) in designing scaffolds which support the formation of complex 3D tissues, many of them with functional vascular networks that match their in vivo counterparts [12,13].

Designing and manufacturing of the scaffold are important areas of biomaterial research for TE and regenerative medicine. [14]. Much work has been done over the past two decades to improve potentially relevant scaffold materials for TE. For neo-tissue generation in vitro and during the initial phase and after implantation, these scaffolds provide mechanical support and encourage cell growth and differentiation [15,16,17]. To date, many materials have predominantly been used to create biodegradable scaffolds comprising polymers with the synthetic origin [18] such as poly(α-hydroxy esters) including poly(ƹ-caprolactone) (PCL), polyglycolic acid (PGA), polylactic acid (PLA), and their copolymer poly(glycolic acid) (PLGA); poly(ethers) containing poly(ethylene oxide) (PEO) and poly(ethylene glycol) (PEG), polyvinyl alcohol (PVA), polyurethane (PU), etc. In addition, naturally occurring biomaterials like polypeptides and polysaccharides are also studied [19]. Composites or blending of these synthetic or natural polymers or together can provide a variety of physicochemical and biological characteristics [20]. Polymer composites, whether natural or synthetic, have some of the most significant applications. A multiphase solid material is a polymer composite in which one phase consists of one, two, or three dimensions in various polymer matrices. Polymer composites are appropriate for use as high-performance composites where the properties of the reinforcement vary significantly from or exceed those of the matrix. In this current review, we are studying polymers (natural and synthetic) as both matrix and reinforcement in a composite. Scaffold materials are defined in terms of mechanical characteristics, chemical composition, and degradation mechanisms. Biomaterial selection plays an important part in the design and production of medical implants and TE products [21]. Although the classical selection criteria for a healthy, durable implant is known as the choice of a passive inert material, any artificial material placed in a patient’s body also generates a cellular response [22]. Therefore, it is now recognized that instead of behaving simply as an inert body, a biomaterial must be biologically suitable and interact with the tissue when implanted [23,24]. In this review, we report on the possible natural and synthetic polymers that have been explored for many years along with their desirable properties and limitations. Besides, the combination of two or more biomaterials, with enhanced functionalities, in the form of either co-polymers, polymer–polymer blends, or composites can satisfy the majority of the clinical requirements by overcoming the limitations of each material. For this, a comprehensive analysis of the recent literature is performed. Many key parameters required for scaffold design, commerciality, and fabrication techniques are discussed in this review.

## 2. Scaffolding and Its Importance in Biomedical Applications (Regenerative Engineering)

**Figure 1:**
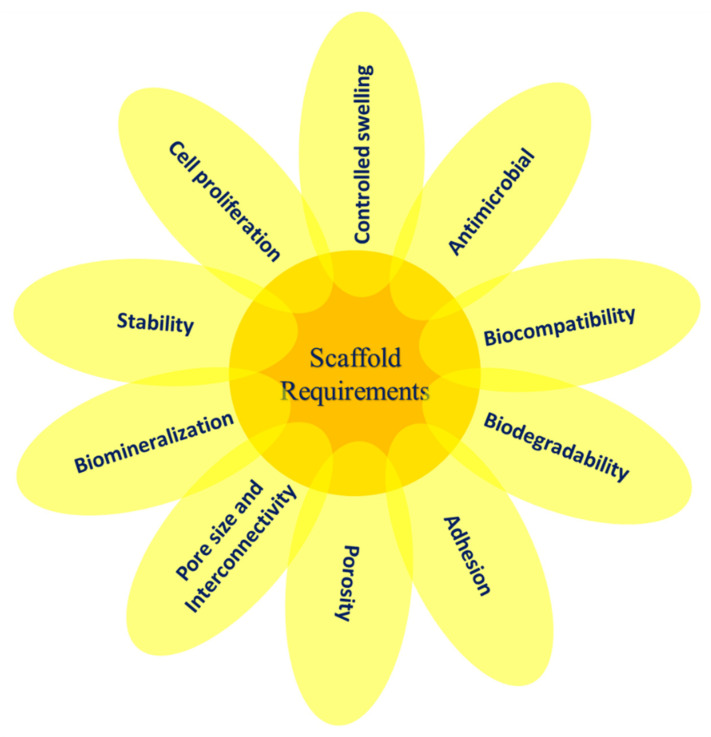
The essential variables involved in scaffold design for TE.

The term “scaffold” refers to an artificial temporary platform applied to support, repair, or to enhance the performance of a structure. This can be done on different size and length scales, with various methods of support depending on the form and use. In general, two-dimensional studies of biomaterial substrates are carried out to test cell–biomaterial interactions. However, to ensure the functions of the damaged tissues, the scaffold is needed to replace the defect or mimic the organs or tissue structures in a three-dimensional manner [25]. Biocompatibility, biodegradability, mechanical characteristics, pore size, porosity, osteoinductivity, osteoconductivity, osteogenesis, and osteointegration are the key design considerations for the scaffold [26,27]. Some of the essentials of scaffolds used in TE are illustrated in Figure 1. After implemented in a body, the scaffold should aim to (i) be a liable structure for adhesion, proliferation, and cell differentiation as a substratum, (ii) create the required biomechanical environment for coordinated regeneration of tissues, (iii) permit the dissemination of nutrients and oxygen, and (iv) allow cells to be encapsulated and released with growth factors [28].

In TE along with regenerative medicine, scaffolds may act as delivery vectors or as cellular systems for drugs and cells. The other choice is to combine scaffolds with different cell types that can enhance osteogenic lineage tissue formation in vivo or release unique soluble lineage molecules. Before being implanted into the target site, these cells can be expanded selectively ex vivo. Scaffolds in clinical medicine are upcoming areas of considerable significance. They are typically associated with organ disease or failure conditions and used to repair organs to restore normal functionality [29,30]. It is well-known that scaffolds support and promote growth of regenerative cells and perform a major role in TE efficiency. Besides, the scaffolding biomaterial facilitates proliferation, differentiation, cell adhesion, offers mass transport and temporary 3D mechanical support, and finally causes the formation of neo-tissue (newly formed tissue built around a scaffold) [31].

In TE applications, the biological crosstalk between the scaffold and the cells is controlled by the properties of the materials and final scaffold characteristics. Materials used for scaffold manufacturing must have intrinsic biofunctionality and appropriate chemistry to stimulate molecular biorecognition from cells to induce proliferation, cell adhesion, and activation. The mechanical properties of the scaffold and kinetics of decomposition in selected materials must be adjusted to the TE application, specifically to ensure the essential structural features and to achieve the rate of new tissue formation. The final effectiveness of the regenerative process plays a major role in scaffolding, exposed surface area, pore distribution, and porosity, the quantity and distribution of which affect the rate of cell penetration within the scaffold volume and the architecture of the extracellular matrix (ECM) formed [23,32,33,34].

Scaffold design for tissue engineering includes several specifications. Many of these parameters are dynamic and not yet well-comprehended. Besides, these scaffolds should possess sufficient mechanical properties to provide neo-tissues with the necessary stress environment. To enable the entrance of nutrients into cells, the scaffolds should be porous, permeable, and have to demonstrate the required surface structure and chemistry for cell attachment [35]. These scaffolds can be created with natural or synthetic polymers or with bio-based ceramics or any suitable combinations.

## 3. Polymers as Biomaterials for Scaffolding

Any substance or a blend of the natural or synthetic source may be used in total or as part of any tissue, organ, or body function to maintain or to enhance, at any time, the person’s quality of life, and then that substitute can be assessed as a biomaterial [36].

In biomedical applications, scaffolds can be used ranging from regenerative engineering to managed drug delivery and immunomodulation; biomaterials have become an indispensable instrument [37]. Regenerative engineering is a multidisciplinary research area that uses the concepts of physics, stem cell science, advanced materials science, clinical translation, and developmental biology for damaged tissue regeneration [38,39].

While several biodegradable polymers are established for use as biomaterials, careful consideration of the particular cellular environment and interactions desired is essential in selecting a polymer for a given application. Applications of this type may include [40]:

- Support for new tissue growth.
- Prevention of cellular activity.
- Guided tissue response.
- Improvement of cell connection and consequent cellular activation.
- Inhibition of cellular attachment and/or activation.
- Prevention of a biological response.

**Figure 2:**
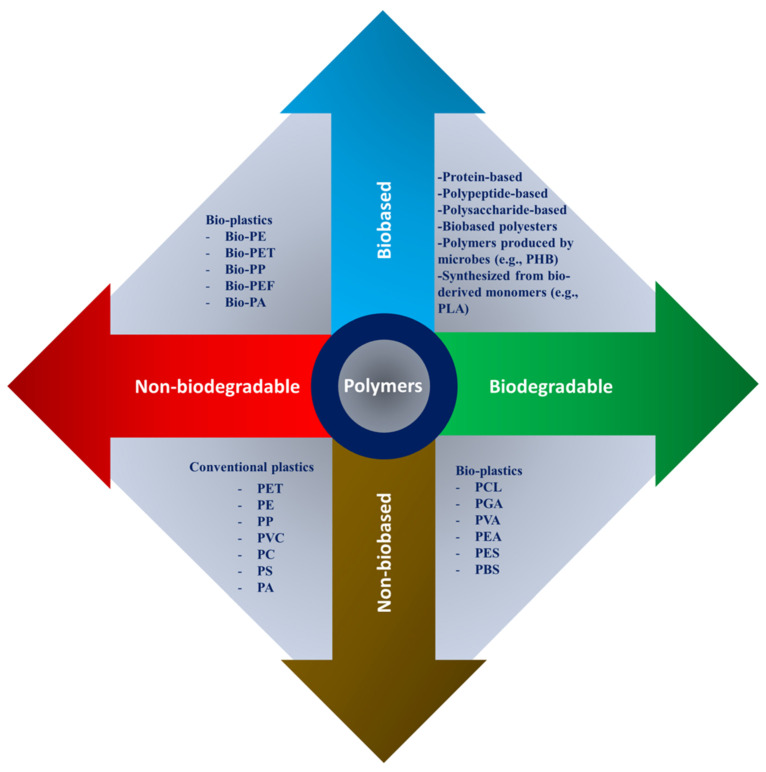
Natural and synthetic polymers were rearranged based on bio vs non-bio and biodegradable vs nonbiodegradable characteristics, where PHB: polyhydroxybutyrate; PLA: polylactic acid; PCL: polycaprolactone; PGA: poly(glycolic acid); PVA: poly(vinyl alcohol); PEA: poly(ethylene adipate); PES: polyethersulfone; PBS: polybutylene succinate; PET: polyethylene terephthalate; PE: polyethylene; PP: polypropylene; PVC: polyvinyl chloride; PC: polycarbonate; PS: polystyrene; PA: polyamide; and PEF: polyethylene furanoate.

Depending on the intended application, scaffold materials can be natural or synthetic, degradable or nondegradable. The polymer’s properties depend on their constituent macromolecules’ structure, composition, and arrangement. The principal forms of polymers used as biomaterials are biologically natural polymers, synthetic biodegradable and nonbiodegradable polymers as shown in Figure 2. Because of their specific characteristics, such as a wide range of biodegradation rates, high porosity with various pore sizes, high surface-to-volume ratio, and mechanical property, polymeric scaffolds attract great interest. They offer distinct benefits of biofunctionality, flexibility, and biological properties that are essential in TE and biomedical applications [31,41,42,43].

### 3.1. Natural Biopolymer-Based Scaffolds

Natural biopolymers have resurged over the past few decades as primary bioactive substances used in the applications of medical materials. Based on their monomeric units and structure, biopolymers are categorized roughly into three classes [27,44]:

- Polypeptide- and protein-based: collagen, fibrin, fibrinogen, gelatin, silk, elastin, myosin, keratin, and actin.
- Polysaccharide-based: chitin, chitosan, alginate, hyaluronic acid, cellulose, agarose, dextran, and glycosaminoglycans.
- Polynucleotide-based: DNA, linear plasmid DNA, and RNA.

These consist of long chains, including nucleotides, amino acids, or monosaccharides made of repeating covalently bonded groups. Biofunctional molecules which ensure bioactivity, biomimetic nature, and natural restructuring are typically found in such polymers. Bioactivity, biocompatibility, 3D geometry, antigenicity, non-toxic byproducts of biodegradation, and intrinsic structural resemblance are the most important properties of natural polymers [38]. Conversely, their key disadvantages, microbial contamination (i.e., endotoxins), decreased tunability, immunogenic reaction, uncontrollable rate of degradation, and poor mechanical strength restrict their application for hard tissue regeneration. Natural polymers make important contributions to TE, especially in the manufacture of scaffolds for therapeutic agent delivery. Novel and natural polymeric materials are aimed at enhancing different therapies due to their inherent bioactivity, biocompatibility, and bioresorbability [31,45]. Naturally derived polymers including collagen, chitin, chitosan, gelatin, silk fibroin, soybean, fibrinogen (Fbg), fibrin (Fbn), elastin, proteoglycan, hyaluronan, and laminin have displayed great potential in the biomedical sector.

#### 3.1.1. Polypeptide- and Protein-Based Scaffolds

Peptides and proteins are polymers that are derived from naturally occurring α-L-amino acids. Peptides are typically shorter (≤100 amino acids) chains, whereas proteins contain longer (≥100 amino acids) chains. In all living systems, proteins are important macromolecules, from bacteria to higher vertebrates, and in mammals, they are estimated to comprise over 50% of their dry cell weight [46,47]. Amino acids are linked via hydrolytically stable amide bonds and are generally degraded by an enzymatic reaction. The lack of processability is the biggest drawback for using them as preliminary materials for commercial biomedical implants. Intrinsic immunogenicity is another constraint of peptide- and protein-based materials towards biomedical applications including scaffold materials for TE. Any peptide- or protein-based polymer brings the possibility that the patient’s immune system may perceive it as a foreign body and cause an immunogenic response. On the other hand, these polymers show outstanding biological properties and can encourage the design of biomaterials with desirable biological activity. This has been the catalyst behind the long-standing curiosity in the use of these materials for TE products for medical implants. Discouragingly, most of the peptides and protein polymers have mechanical properties that are not conducive for the use of medical implants which require mechanical strength, such as scaffolds for bone regeneration, thus limiting their practical applications [48,49,50,51].

Collagen is a primary structural element of the native ECM and has several functional characteristics that help to bind the cell, proliferation, differentiation, and secretion of the ECM. Collagen scaffolds are important biomaterials used for TE for reconstruction of many forms of tissues and organs. While a few of its uses are incredibly successful and are now implemented for clinical treatments, some are still in the preliminary phase. Controlling biodegradation and improvement of their mechanical properties remains a challenge. Contraction and deformation of collagen-based scaffolds have limited their application to load-bearing tissues [52]. To date, more than 20 different members have been identified in the collagen superfamily. Among all the members, right triple helix made up of three α-chains is one of the characteristic structural features. These may consist of three identical chains (homotrimers) as in collagen types II, III, VII, VIII, and X or of two or more different chains (heterotrimers) as in collagen types I, IV, V, VI, IX, and XI. With a pitch of 18 amino acids per turn, each of the three alpha-chains inside the molecule forms an expanded left-handed helix. Type I collagen is an attractive medium for further advancement of TE scaffolds, considering its proven clinical effectiveness for short- and medium-term usage and possible smooth access to the health products market. However, its restricted chondrogenic capacity, poor mechanical strength [53], and substantial shrinking [54] can impede the long-lasting clinical effectiveness of type I collagen scaffolds. Type II collagen-based scaffolds are a very good substitute to type I collagen if chondrogen output is considered. It is generally applied in cartilage regeneration owing to its inherent flexibility, but additional studies are needed to validate safety problems for type II collagen [55,56]. In the body, collagen degradation is caused by the existence of enzymes such as collagenases and metalloproteinases that produce subsequent amino acids. Collagen scaffold composition may be modified to achieve improved biological activity and mechanical properties of the final scaffold by combining with other molecules such as hyaluronic acid (HA), chitosan, and chondroitin sulphate (CS) [57].

Gelatin is the result of degradation derived from insoluble collagen by disintegration or denaturation. Though collagen comes in several types, gelatin only comes from alkaline or acidic hydrolysis of type I collagen. Generally, it can be extracted from animal collagen, bones, skins, and tendons, either with partial acid (type A gelatin) or alkaline hydrolysis (type B gelatin). The isoelectric point of type A gelatin is identical to collagen. The isoelectric point relies on the collagen extraction process and changes to permit gelatin to bind with either positively or negatively charged therapeutic agents. Type A gelatin with an isoelectric point of 5.0 could be used in vivo as a carrier for basic proteins, whereas type B gelatin with an isoelectric point of 9.0 could be used in physiological conditions for the continuous release of acidic materials. It has complex physical characteristics and chemical heterogeneity due to discrepancies in collagen sources and preparation techniques [58]. Gelatin comprises 19 amino acids connected in a partially organized manner and has a polyampholyte surface property. However, gelatin is negative at higher pH and positive at lower pH [59]. Gelatin polymer is primarily limited by its high biodegradation rate due to enzyme digestion and high physiological solubility which describes its low mechanical stability, leading to a disparity between the new formation of bone and the degeneration of scaffolds. Alongside this, the role of higher-order gelatin structures and bioactivity of scaffolds is still raising many unanswered questions. The most important thing is whether the cells are sensitive to the secondary and higher-order gelatin structures in the scaffolds [60]. At the same time, due to the existence of active chemical groups (e.g., NH_2_ and COOH), chemical treatments may be performed to increase the degradation period [61].

Silk is a natural protein-based polymer derived from various Lepidoptera larvae, such as spiders, as well as silkworms. In nature, silk displays numerous combinations, structures, and functions. This complex behavior of silk is induced by its source and atmosphere. Silk fiber is a perfect blend of high strength, low weight, excellent durability, and elasticity (the strongest natural fiber). Silk is made up of two separate main proteins; one is silk fibroin (SF) made from the fibrous portion of the filament and another is sericin, which is a glue-like and water-soluble protein containing 18 different kinds of amino acids [62]. Studies have shown that, in addition to a tenable degradation rate and mechanical properties, the manufacturing of silk fibroin-based scaffolds of varying configurations yields attractive biocompatibility. Fibroin has been shown to have predictable proteolytic degradation in comparison to other biological materials by modifying fibroin diameter, several failure intervals, failure strength, and mass degradation [63]. Apart from the extraordinary mechanical properties, silk is biocompatible, thermostable (up to ~250 °C), and processable in a wide range of temperatures. Several scientists have tested composite silk scaffolding to achieve the necessary characteristics by changing the blended materials, the silk-producing source, or the material concentration in that composite, and it was proven very suitable for the TE. [64]. The versatility of silk fibroin can be seen in its various applications, from silk as a bulk part to silk as a coating or reinforcement of non-cytocompatible scaffolds. Silk fiber inclusion increases the compression strength in both in vivo and in vitro tests, minimizing setting time without adverse impact on injectability and cytocompatibility. Silk fibroin is one of the products that are favorable for bone tissue scaffolding applications due to its specific moderate mechanical properties, more controllable degradation rate than of many natural polymers, and high biological compatibility [62].

Fibrinogenic and fibrin-based scaffolds can provide an adequate environment for the natural matrix. Provided that these primary materials are available widely as the main coagulation proteins in blood, native biochemical associations with damaged tissues and cells can be easily communicated [65]. Similar to collagen scaffolds, fibrinogenic and fibrin-based scaffolds may achieve high efficiency of cell seeding and uniform cell distribution by proliferating, migrating, and differentiating into specific tissues/organs through the secretion of the ECM. There are also several drawbacks such as weak mechanical properties for the regeneration of skeletal tissues, the potential for transmission of diseases through unpredictable biological affinities, and fibrin deformation [66]. Fibrinogen provides significant healing benefits as it provides an attractive proliferation surface, cellular attachment, 3D fibrous structural support, and nanotextured surfaces consisting of a fibrous cell signaling network and cell–cell interactions. [67]. Fibrinogen- and fibrin-based scaffolds induce ECM development in TE for supporting connective tissues like nerves, blood vessels, skin, ligaments, bones, cartilages, and tendons. Fibrin-based scaffolds promote and offer enough time for neo-matrix development while resorbing gradually due to the action of proteases. These therapeutic assets facilitate wound healing and reduce the formation of scars for more natural, functional, and esthetic characteristics [68].

#### 3.1.2. Polysaccharide-Based Scaffolds

Another group of naturally occurring polymers is polysaccharides made of different units of monosaccharide or disaccharide chains (e.g., starch, cellulose, etc.). The effect is an incredibly large number of structurally diverse polysaccharides as numerous distinct saccharide isomers are mixed by utilizing a range of chemical bonds. The polysaccharide chemistry is as rich as of proteins in terms of diversity and heterogeneity. Therefore, it is not unexpected that different saccharides and polysaccharides perform a major role in finely tuning cell environmental response [69]. It is possible to categorize polysaccharides into structural and storage polysaccharides. Cellulose in plants and chitin in crustacean shells are examples of structural polysaccharides, while starch and glycogen can be included in storage polysaccharides [70]. Despite these benefits, there are some restrictions on the use of natural polysaccharides to prepare scaffolds. Their distribution, branching, and sequence of molecular weight are not consistent. These differences may be deleterious for biorecognition events as well as affect rheology. Generally, many naturally occurring polysaccharides are not biodegradable when introduced in mammalian species because of the absence of digestive enzymes. As a result, these are not a primary material option in the biomedical application without further chemical alteration [71].

Chitin and chitosan are interesting materials for biomedical and pharmaceutical applications because they have positive properties that make them ideal in the biomedical field, such as non-toxicity, biodegradability, and biocompatibility [72]. These materials often reflect a wide range of proprieties owing to their reactive hydroxy and amino groups, high charge density, as well as their broad hydrogen-bonding capacities and the single chemical structure. The combination of diverse physicochemical and biological features allows a vast variety of biomedical uses [73]. Chitin is generally found in shells of crustaceans and its derivative chitosan is obtained by deacetylation of chitin. These are glycosaminoglycan-like natural cationic polysaccharides [74]. Applicability of chitosan includes implantable and injectable orthopedic and periodontal devices, wound healing agents, lung surfactant additives, drug delivery systems, and TE scaffolds due to its high biodegradability and biocompatibility along with its unique interactions with ECM components and growth factors [75]. Owing to the excess of their reactive amino and hydroxy groups and cations, chitin and chitosan are coupled with other molecules to boost the biological functions of other materials in implant products. For instance, it is established that the hydrophilicity of other biomaterials and their biocompatibility are improved by chitosan coating. These chitosan-coated composites can promote cell proliferation and adherence [76,77]. The key route for the in vivo breakdown of chitin and chitosan is known to be lysozymes which slowly act to depolymerize the polysaccharide. The biodegradation rate depends on the acetyl content quantity, which is an easily variable parameter. Chitin and chitosan modification producing significant products with enhanced properties as required for scaffolds has been explored, and research in this field of biomaterials will continue to be pursued [78].

Hyaluronic acid (HA) is a linear polysaccharide, ubiquitous and extremely biologically compatible in the ECM of mammals. HA is a glycosaminoglycan found in many areas of the body in the extracellular tissue [79,80]. It is an increasingly important material for the study of biomaterials and finds applications in different fields stretching from tissue culture scaffolds to cosmetic materials. Its physical and biochemical properties both in the solution and hydrogel forms are highly desirable to different body repair technologies [81]. HA is an essential part of connective tissue where it plays a major role in cell growth, cell differentiation, and lubrication. HA includes functional groups such as carboxylic acids and alcohols that can be used for the implementation of functional domains or the development of a hydrogel by connecting them. HA can form a new type of TE scaffold which is both bioactive and biodegradable. It shows low non-specific protein adsorption and can be tailored to facilitate growth and repair of tissues via cell receptors [82].

### 3.2. Synthetic Biopolymer-Based Scaffolds

Synthetic polymers are advantageous in a few characteristics such as tunable properties, endless forms, and established structures over natural polymers. The support offered by synthetic biomaterials can enable restoration of damaged or diseased tissue structure and function. Polymerization, interlinkage, and functionality (changed by block structures, by combining them, by copolymerization) of their molecular weight, molecular structure, physical and chemical features make them easily synthesized as compared to naturally occurring polymers [83,84]. The disadvantages of synthetic biomaterials are that they lack cell adhesion sites and require chemical modifications to enhance cell adhesion. Many commercially available synthetic polymers exhibit similar physicochemical and mechanical characteristics to biological tissues. In biodegradable polymers, synthetic polymers are a major category and can be produced under controlled conditions. In a broad spectrum, the mechanical and physical characteristics are predictable and reproducible, such as strength, Young’s modulus, and degradation rate. Poly(α-hydroxy esters) including PCL, PGA, PLA, and their copolymer PLGA and poly(ethers) including PEO and PEG, PVA, and PU are the most widely studied degradable synthetic materials. These are probably the most popular examples, although there are currently many other synthetic materials being sought [85,86,87]. These polymers have various levels of biodegradability, biocompatibility, and mechanical properties, but no single polymer holds all three of these critical properties at the optimum level [88].

PLA is a gradually crystallizing semicrystalline polymer [89]. Due to its host tissue biocompatibility, hydrophobic nature, relatively simple processability, and biodegradability, PLA is one of the unsurpassed choices for numerous biomedical applications without the need for a second intervention [90,91]. PLA is largely prepared from the lactic acid (LA) monomer through the fermentation process of natural resources such as wheat and grain or by various routes of polymerization as a petrochemical derivative. PLA degradation products, specifically, water and CO_2_, are neither carcinogenic to the human body nor harmful [92]. This substance can be available in many forms, for example, as poly(L-lactic acid) (PLLA), poly(D,L-lactic acid) (PDLA), and poly(D,L-lactic acid) (PDLLA), that can be used for various tenacities, such as for the manufacture of screws, pins, rods, plates, including for biomedical implants, and is suitable for multiple purposes [93]. The easiest linear aliphatic polyester is polyglycolic acid (PGA). It is not soluble in the majority of organic solvents due to its high degree of crystallinity. By random cleavage of its ester linkages in the backbone, it undergoes bulk erosion. Under physiological conditions, PGA breaks down into glycolic acids that can join the tricarboxylic acid cycle and be expelled from the body as water and CO_2_ [94]. Polylactic-co-glycolic acid (PLGA) is known as a random ring-opening copolymer of PLA and PGA. PLGA is a biodegradable polymer thanks to its non-toxicity, high cell adhesion, controllable degradation rates, and favorable mechanical properties [95]. The pendant methyl side group on the structure of the PLGA chain causes the hydrophobic surface similar to PLA [96]. In this regard, the degradation rate of PLGA products can be regulated by varying the percentage of these two polymers [97,98].

In TE, PLA, PGA, and their copolymer PLGA are commonly used to treat patients with organs or tissues that have been damaged or destroyed. They have demonstrated their biocompatibility, their deterioration into non-toxic products, and a long history of use in degradable surgical sutures [99]. PLA, PGA, and PLGA degrade via hydrolysis of ester bonds. When degraded, natural pathways eliminate the monomeric components. The body includes highly regulated pathways to fully eliminate lactic and glycolic acid monomeric components. While PGA is converted or removed by other pathways into metabolites, PLA is cleared through the cycle of tricarboxylic acid. Because of these properties, PLA and PGA are used in products and devices approved by the US FDA, including in degradable sutures [99]. PLA and PGA can be simply processed and their physical and mechanical properties and degradation rates can be modified using different molecular weights and compositions of copolymers over a wide range [100,101].

PCL is a semicrystalline and aliphatic polymer that is extremely tough and demonstrates sufficient biocompatibility. PCL’s hydrophobic nature prevents cell adhesion and cell proliferation [102]. Initially, PCL degradation takes place in amorphous substance domains, which means that crystal dominances remain untouched. At this stage, non-enzymatic bulk hydrolysis of ester connections catalyzed by the carboxylic acid end groups is carried out [103]. A foreign body response consisting of giant cells and macrophages with a few neutrophils occurs after the material becomes very brittle with extensive hydrolysis [104]. Copolymerization, surface functionalization, or blend formulation are some of the approaches to enhance its bioactivity. The rate of deterioration is relatively slow (2–4 years) and it is degraded by the hydrolysis of its ester linkages under physiological conditions [105].

Polyethylene glycol (PEG)-based polymers, in terms of biomedical applications, are non-ionic, biocompatible, and have optimal physicochemical and biological properties. After implantation, PEG is minimally immunogenic. A variety of cross-link methods are used to manufacture hydrophilic PEG scaffolds. The selected cross-linking process can affect the scaffold’s physiochemical characteristics, including permeability, molecular diffusion, elasticity, modulus, or rate of degradation [106]. Poly(ethylene oxide) (PEO) is a hydrophilic polymer that is usually inert with minimal antigenicity, immunogenicity, cell adhesion, and protein binding [107]. Its inhibition of binding proteins is caused by a lack of groups contributing hydrogen. In the 1970s, PEG, the shorter molecular form of PEO, became famous when scientists discovered that the polymer inhibits absorption of proteins. The photopolymerization capabilities of both PEO and PEG include adaptable mechanical features, as well as simple control of the architecture of scaffolds and chemical composition, which all make them appealing scaffold materials for the creation of 3D tissue regeneration templates [108].

Another cross-linkable, biodegradable, high-strength polymeric biomaterial engineered for orthopedic applications is co-polyester poly(propylene) fumarate (PPF). It is a linear polyester with repeating units containing ester bonds [109]. The double bonds of fumarate in PPF can be crosslinked to form polymer networks at low temperatures. They are particularly suitable for orthopedic applications because of high mechanical strength. The ester bond hydrolysis allows PPF to degrade, and degradation time can be affected by several factors (such as molecular weight, curing agent types, and cross-link density) [110]. In the presence of water, PPF degrades into propylene glycol and fumaric acid, products of degradation that are quickly removed from the human body by natural metabolic processes. PPF is commonly used to improve PLA, PGA, or PCL hydrophobicity.

Polyurethane (PU) contains a urethane moiety in its repeating units. The reaction of diisocyanate with polyol normally produces these polymers. In the manufacture of blood-contacting devices such as artificial veins and arteries or heart valves, polyurethanes are the most widely used materials, and have also been used to engineer tissues such as bones, heart muscles, heart valves, blood vessels, skin, skeletal muscles, and cartilages [111]. They provide a large family of materials with the only common feature of urethane links in large molecular chains. Urethane links typically formed by isocyanate and alcohol reactions. In the preparation and treatment of polyurethanes, in addition to the formation of urethane bonds, several other reactions lead to the development of various bonds such as allophane, biuret, acyl urea, or isocyanurate, which may result in further branching or crosslinking affecting the overall physical and chemical properties and biocompatibility [112,113]. Alternative diissocyanin compounds are required to design biodegradable polyurethanes because conventional aromatic diisocyanate is toxic and presumed carcinogenic. Biodegradable diisocyanates, such as lysine diisocyanate or hexamethylene diisocyanate, release non-toxic products while degrading [114].

In general, synthetic polymers, when used as a scaffold, PGA and its copolymers, such as PLGA, degrade too rapidly since their tensile strength decreases by half within two weeks. PLLA, on the other hand, degrades too slowly, taking about 3–6 years for maximum resorption. In recent tissue engineering studies, lactide copolymers, such as lactide-ε-caprolactone copolymers (LA-CL cop), were given preference because of this unsatisfactory resorption property of PGA and PLLA. According to various studies, the degradation rates of some synthetic biodegradable polymers decrease in the following order: PGA~PLGA > PDLLA > PLLA > PCL~PPF > polybutylene succinate (PBS). When comparing, the degradation rate of PCL happens at a considerably slower pace than that of PLA, PGA, and PLGA. This slow degradation makes PCL less attractive for soft tissue engineering applications but is more desirable for long-term implants and controlled release applications [8,13,115] such as bone TE where mechanical and other physical properties should be maintained for at least 6 months by scaffolding. On the other hand, PPF and other biodegradable polymers lack the mechanical strength that is needed for load-bearing applications such as bone TE [116,117,118,119].

While some of the polymer scaffolds mentioned above degrade enzymatically and/or hydrolytically, several other polymers, including PEO, PEG (i.e., the low molecular weight variant of PEO), and PVA, are used to induce more rapidly degrading or instantly soluble characteristics in scaffold materials [85,87]. The mechanical characteristics of PLA differ depending on their molecular weight and optical purity. High molecular weight increases the Young’s modulus and strength and decreases the elongation at break. Regularly used PLA (approximately 5% d-lactic acid) is a brittle material, with little elongation at break (6% to 11%), 900–1300 MPa elastic modulus, and 61–73 MPa strength. In contrast to PLLA and PDLA, PLA has great mechanical characteristics [120]. PLA has mechanical properties identical to polyethylene terephthalate (PET), but the maximum continuous usage temperature is significantly lower. Polyhydroxybutyrate (PHB) has little elongation, is brittle, and quickly tears under mechanical pressure [121].

Synthetic polymers are produced from hydrocarbon building blocks in the laboratory setting. Although the intrinsic cell interaction moieties of the biopolymers may be lacking, their capacity to be specifically controlled in structure and reproducibility make them useful along with natural polymers in biomaterial composites for TE applications [122,123].

A variety of polymeric substances used for biomedical scaffolding applications particularly towards regenerative engineering, both naturally and synthetically fabricated, are tabulated in Table 1 along with their advantages and disadvantages.

### 3.3. Natural–Natural Biopolymer Composites

As such, because of the broad disadvantage of allografts and alloplastic implants, including the lack of donors for donating tissues and organs, the possibility of immunological transplant rejection, tiredness, and fatigue, TE offers the opportunity to rebuild damaged or destroyed tissues without any complications [45,78,175]. The particular interest is the natural biological macromolecules due to their excellent biocompatibility, low immunogenicity, and cytocompatibility, as well as the antigenic nature that makes them popular for TE scaffolding applications [31].

Polymer mixes describe a polymer material consisting of at least two or more polymers resulting in improved physicochemical properties compared to different individual polymers. Each one of the polymers holds its particular biological and physicochemical properties in a blend. It allows improving strength and rigidity while ensuring low density and lower weight compared to monocomponent polymers [41]. Though many studies stated their minimal mechanical effectiveness and superior sensitivity to environmental factors, such as temperature and humidity, blend growth is the major downside of bulk natural polymers [31]. Some of the natural biopolymer blends are tabulated in Table 2.

### 3.4. Natural–Synthetic Biopolymer Composites

There are major advantages to natural biopolymers over synthetic materials, including lower/no toxicity, better bioactivity, enhanced cell response when associated with cells, excellent biocompatibility, extreme hydrophilicity, and effective biological function. However, their weak engineering properties often limit the utility of natural biopolymers. Significant drawbacks of natural biopolymers are as follows:

- High batch-to-batch inconsistency owing to complicated isolation techniques from inconsistent sources.
- Poor processability and solubility blocking the utilization of industrial fabrication processes.
- Possibility of contamination by pyrogens and pathogens.
- Poor or limited material properties like elasticity, ductility, strength, and shelf life.
- High cost.

In contrast, synthetic polymers have shown many advantages, improved control over chemical composition, especially in terms of processability and good mechanical properties, but in scaffold products, there is insufficient bioactivity, low cell attachment capacity, hydrophobicity, and limited surface cell recognition.

It is proposed that not one substance gathers all the criteria for tissue replacement. Instead, a scaffold made from a composite containing both natural and synthetic biopolymers can provide a tissue substitute that satisfies all clinical requirements comprising the specific size and kind of wound, the age of the patient, and the procedure of preparation available [123]. Several researchers have investigated the use of a particular combination of natural and synthetic materials for manufacturing tissue scaffolds to take advantage of the intrinsic biocompatibility of natural materials and the physicochemical properties of synthetic polymers. A few natural–synthetic polymer composites along with their properties, biological assessment, and characteristics are tabulated in Table 3. Greater control of degradation rate is made possible with the use of different synthetic vs. natural product formulations [123]. The combination of natural and synthetic polymers (bioartificial combination) is a multipurpose method to design more successful biomaterials that enhance physical and biological features (for example, biocompatibility) [31]. They have been combined to take advantage of their favorable properties to overcome the disadvantages of each particular type of material [186].

## 4. Properties of Polymer Scaffolds

When dealing with the polymers for scaffolding materials, the properties of the polymer can be classified into three groups based on their intrinsic nature, processing conditions, and final product. The intrinsic properties are inherent characteristics of the polymer itself that depend primarily on its chemical structure and composition (e.g., crystallinity, density, solubility, transition temperatures, mechanical, electromagnetic, gas barrier properties, transparency, etc.) [201]. Processing characteristics include the melt flow index, viscosity, and the strength of the melt. While these properties suggest the material’s behavior during the forming process (e.g., in extrusion), the practitioner requires additional details like the working conditions of each material at the different production stages. In general, the properties of the product are defined by combinations of both intrinsic properties, mechanical behavior, water resistance, heat resistance, esthetic properties, and environmental behavior including degradation conditions [202,203].

The structures should fulfill certain criteria that are necessary for use as scaffolds in TE. The key constraints to consider when constructing a scaffold are biological properties and physical properties. According to the tissue characteristics, certain properties can differentiate the right material, and a processing method must be chosen to manufacture these scaffolds with different characteristics. Some of the desired properties are biocompatibility, biodegradability, morphology, pore size and porosity, and mechanical strength [204].

### 4.1. Bioactivity

Bioactivity refers to a material’s ability to impact its biological surroundings. Since the invention of ”tissue engineering”, [205] biomaterials have historically been used to offer a bioactive environment, in which cells adhere and propagate [206]. Three-dimensional (3D) scaffolds may actively interact with cellular components of the engineered tissues to promote and control their activities. Biomaterials may contain biological signs such as cell-adhesive ligands to reinforce attachment or physical signs like topography to influence cell morphology and orientation [207]. The scaffold may also act as a delivery vehicle or a repository for exogenous growth-stimulating signals like growth factors to speed up regeneration. In this regard, biomaterials must be compatible with biomolecules for the controlled release of bioactivity-retained biomolecules and compatible with the encapsulation technique.

### 4.2. Biocompatibility

Biocompatibility is one of the essential attributes to be considered when designing soft and hard tissue scaffolds [92,208]. Biocompatibility describes the capacity of a biomaterial to execute its intended purpose concerning medical therapy without affecting the client or beneficiary of that therapy from suffering any adverse local or systemic effects. In that case, it should produce the most suitable cellular or tissue response and maximize the clinically relevant efficiency of that therapy. Molecular processes should not be poisonous, causing the host tissue to be immunologically rejected. As part of the protective system, the body develops immunological responses to foreign substances entering the body and triggers the rejection of scaffolds or implants. Therefore, only negligent immune reaction should be triggered by any bioengineered structures, so that the inflammation caused by this does not hinder the healing process or trigger any in vivo toxicity [204]. Besides, the structure must have biomimetic binding sites to conform to the cells, which can contribute to proliferation and differentiation.

**Figure 3:**
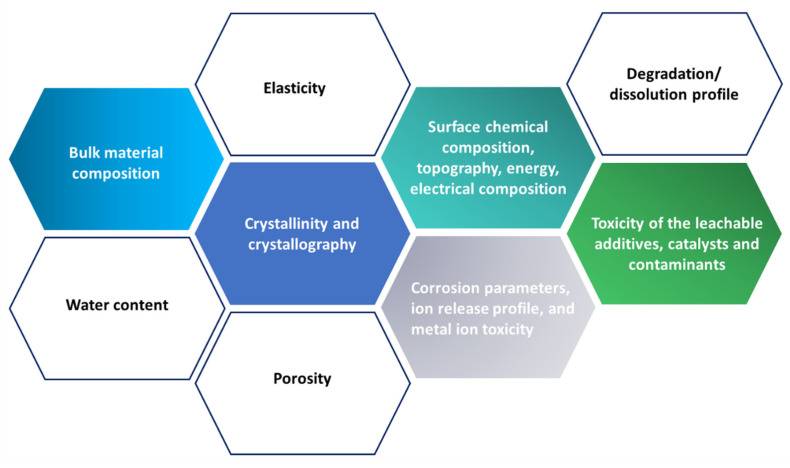
The essential variables that define the scaffold’s biocompatibility.

Some main factors which define biocompatibility of the scaffolds are shown in Figure 3. Adsorption and desorption activities of polymeric materials of various types of mammalian cells depend on surface characteristics such as surface roughness, rigidity, hydrophilicity/hydrophobicity ratio, bulk chemistry, surface charge, and charge distribution. In order to improve biocompatibility of surfaces in contact with living tissue, a wide range of surface treatments are available to seal unwanted residues or additives by means of a coating and to control excretion and/or absorption using a selectively permeable surface.

### 4.3. Biodegradability

Excellent biocompatibility over time is essential for biomaterials that are biodegradable, as the physicochemical, mechanical, and biological characteristics of a biodegradable material change over time, and degradation products that are tissue-compatible to the original material vary. Degradation materials that are non-toxic and quickly metabolized and cleared from the body should provide an ideal biodegradable biomaterial [209]. The scaffold’s absorption kinetics is essential and depend on the regenerating tissue. If a scaffold is employed for skeletal system TE, the biomaterial deterioration of the scaffold can be relatively gradual, as the mechanical strength must be preserved before the tissue reconstruction is nearly complete. In contrast, the scaffold does not need to last more than one month for skin TE. If the scaffold leftovers remain in the body for a longer period than needed, the residual material can retard rather than facilitate the regeneration of the tissue.

The gradual breakdown of a material mediated by a specific biological activity applies to the term biodegradation. More specifically, if this breakdown is due to cells and/or tissue activity, the substance should be described as biodegradable. The term “biodegradation” is often used to describe materials which are less independent of the degradation process after implantation in a bodily location. However, the fact that biodegradation products are metabolized or removed from the body is essential.

Due to their improved overall interactions with different cell types and lower or lack of immune response, natural polymers were among the first biodegradable scaffolding agents to be used clinically. However, despite the possibility for immune response or toxicity, synthetic polymers were later found to be cheaper and more effective than natural polymers, particularly, several polymer combinations [206].

Scaffolds can be classified into two types based on their degradation property, permanent/nondegradable and biodegradable. A permanent scaffold must not decay and the properties of the soft tissue it substitutes should be consistent [204]. However, the important factor requiring an in vivo analysis is the rate of biodegradation. The rate of biodegradation of a polymer is mostly contingent on the polymer’s intrinsic properties, including the chemical structure, hydrophilicity/hydrophobicity level, crystalline/amorphous morphology, the existence of hydrolytically unstable bonds, glass transition temperatures (T_g_), molecular weight, and copolymer ratio, so that a wide variety of maxillofacial applications can be manipulated within a week or months or years. The byproducts, without inducing any cytotoxicity, can easily exit the body. For inflammatory responses, the regulated activity of macrophages is required so that degradation can happen beside the growth of a new tissue. After its function is served, a biodegradable scaffold is required to degrade on its own in order to replace it with new cell growth. When constructing a scaffold, degradation rate and the degradation mechanism are important factors to be studied [210].

The degradation of scaffolds can happen via mechanisms involving physical or chemical processes and/or biological procedures that are intermediated by biological agents, such as tissue remodeling enzymes. Based on their method of preparation, biodegradable polymers are categorized into two main classes: stepwise polycondensation or ring-opening polymerization. Agro-polymers such as polysaccharides and proteins comprise the first group [211]. Biodegradable polyesters, including aliphatic polyesters and aromatic polyesters, are in the second group. Biodegradable polymers have a wide variety of properties and can be substituted for nonbiodegradable polymers in a variety of applications, such as biomedical, textile, and packaging applications [208].

In biodegradable polymers, non-biological processes such as hydrolysis and erosion or biological processes such as enzymatic action or intervention by microorganisms such as bacteria, yeast, and fungi may initiate the degradation process. The majority of natural polymers have been documented to be enzymatically degraded. In the case of protein-based biomaterials, enzymes such as collagenases and metalloproteinases degrade peptide bonds in vivo [204]. The biomaterials dependent on polysaccharides are degraded by lysosomes and amylases within the body. On the other hand, most synthetic biodegradable polymers include hydrolyzable linkages that are degraded by a hydrolytic process, such as ester, urea, and urethane linkages. In parallel, these processes act to speed up the degradation of these polymers. The polymers’ hydrophobic/hydrophilic nature substantially changes their biodegradability. In general, polymers with more polar groups are easily biodegradable. The entire process of biodegradation can range from days to months to years depending on the kind of polymer. Cell biomass and other intermediates can inevitably be mineralized to CO_2_ over a long period [212,213].

**Figure 4:**
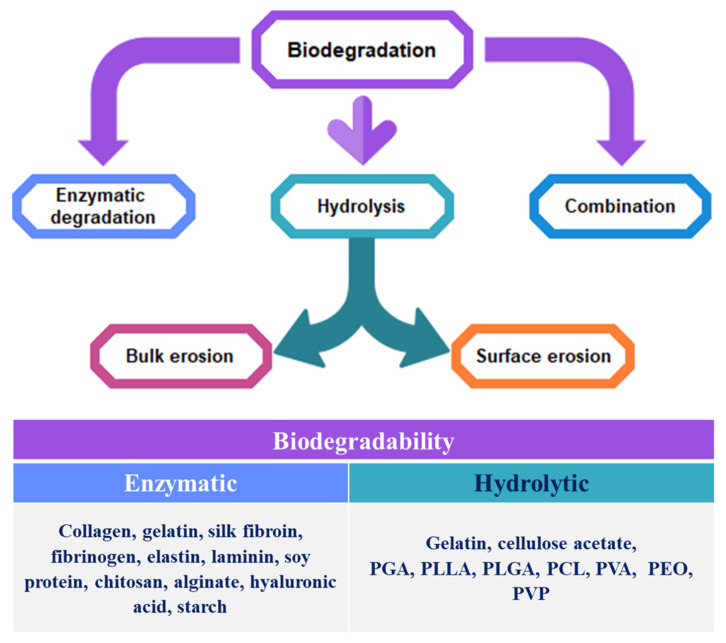
Biodegradation mechanisms of natural and synthetic polymers.

In non-biological processes, chemical splitting is largely responsible for deterioration, along with physical erosion. In semicrystalline biodegradable polymers, amorphous domains are highly susceptible to water molecular diffusion [214]. In this case, the hydrolytic degradation arises first in the polymer’s amorphous regions, heading to chain splitting. After that, the hydrolytic degradation takes place in crystalline parts. It should be noted that the degree of hydrolysis depends significantly on the relative hydrophilicity of the polyesters engaged. Physical erosion is associated with hydrolysis that assists with degradation. In physical erosion, the two processes resulting in the breakdown of the scaffold and resorption/dissolution of the material involved are bulk erosion and surface erosion. Although bulk erosion is associated with mass loss all over the material, the erosion of the surface is restricted only to the particular surfaces subjected and continues via an erosion front. Bulk erosion is prominent in the case of biodegradable aliphatic polyesters, leading to sample fragility and compromising the materials’ mechanical and functional capabilities [214]. Therefore, while the scaffold size turns out to be smaller, the bulk structure is retained. These forms of degrading scaffolds provide the tissue to regenerate with more mechanical stability. The biodegradation behavior of different polymers in both biological process and non-biological process is illustrated in Figure 4.

The cleavage of hydrolytically or enzymatically sensitive bonds in polymers leads to polymer erosion and biomaterials degradation. On the other hand, most synthetic biodegradable polymers contain hydrolyzable linkages that are degraded by a hydrolytic process, such as ester, urea, and urethane linkages [215,216]. Biologically stable, nonbiodegradable polymeric scaffolds can deliver permanent support over time and should work best during the patient’s lifetime. Future research will illustrate several parameters that are needed to optimize and monitor desired applications, such as polymerization conditions, compositions, and scaffolding techniques [208]. Degradation mechanism, duration, and solvent in the decay of some of the biodegradable polymers including both natural and synthetic polymers and their composites are tabulated in Table 4.

### 4.4. Porosity and Pore Size

**Figure 5:**
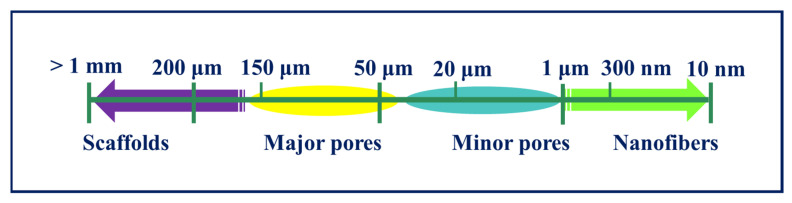
Scheme of different size scales of relevant structures.

The pore size, porosity, and mechanical properties of scaffolds play a vital role in TE. A scaffold’s porosity relies on its pore quantity, pore size, form, connectivity, and orientation. Porosity typically promotes proliferation and migration of cells, delivering an atmosphere for the transmission of nutrients to the structures underlying or near the scaffolds. Porous scaffolds ensure cell growth, uniform distribution of cells, and vascularization [27,234,235]. Some of the polymer scaffold materials with good porosity and biomedical applications are tabulated in Table 5. Scaffolds must have a highly porous structure with an open, completely interconnected geometry. The porosity and, sequentially, the surface-to-volume ratio of the scaffold should not be so high that its mechanical strength is weakened [236]. 3D scaffolds face two major restrictions for TE applications—a scaffold can neither be too porous (due to compromised mechanical strength) nor significantly lack porosity (due to the lack of cellular ingress, vascularization, and signaling) [206]. The major parameters to consider while constructing a scaffold are average pore size, pore size distribution, pore length, pore interconnectivity, pore shape, pore throat size, and pore wall roughness. It establishes a biocompatible porous network from which the surrounding tissue is induced and serves as a temporary model for the development and reorganization of the new tissue. The pore size should not be less than 100 μm in diameter for the full diffusion of oxygen and nutrients to promote cell survival [237,238,239]. However, some specific pore sizes are defined for hard tissues that are in the range of 200–350 μm; some of them can be seen in Figure 5. Furthermore, scaffolds should have a suitable surface area with optimum porosity. Reduction of compressive and tensile properties is one of the drawbacks of increasing porosity [208,219].

### 4.5. Morphology

Both chemical and topographical characteristics are involved in surface properties, which can control and influence cellular adhesion and proliferation. The surface of the scaffold is the original and principal interface site surrounding cells and tissue. Since most cells used for TE are based on anchorage, it has been suggested that their attachment may be facilitated by the scaffold. Therefore, scaffolds with a wide and functional surface area are favorable [246].

Scaffolds should be constructed to be consistent with the structure of the tissue and have a large surface area, high porosity, fully interconnected geometry, structural strength, and a particular three-dimensional shape [247]. Moreover, it has to be biocompatible in order to enable long-term substitution for a newly developed tissue. A given structure matrix design with particular material properties is necessary for every tissue. Along with the size of the pores, the performance of the implanted matrix and the rate of tissue ingrowth can be significantly affected by morphology [248]. The optimum porosity is strictly linked to the type of tissue, and a different microenvironment can be associated with diverse tissue architectures. Cell dimensions must be considered when designing a scaffold for TE, together with phenotypic expression, cell activity, and ECM production [247].

### 4.6. Mechanical Properties

**Figure 6:**
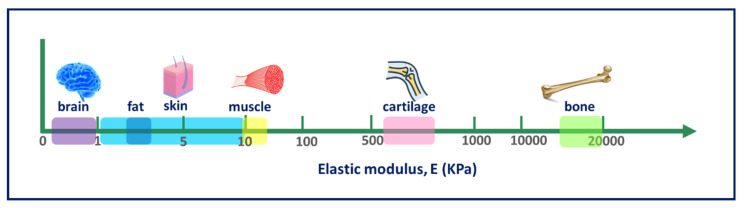
Schematic depicting the normal variation in elasticity of the indicated tissue.

To preserve integrity of the scaffold during implantation, mechanical strength is determined by the impact resistance of the final goods. Tensile and compressive tests include the most common mechanical tests to assess scaffolds. For the performance of the implant, the sufficient mechanical properties for a biomaterial to be used in a TE application are important. Factors such as elasticity, strength, and absorption at the material interface and their degradation depend on the biostability of several scaffolds and are important. In general, biopolymer scaffolds should have the mechanical properties appropriate for the site of implantation and should have the strength that is needed for any implantation requiring a surgical procedure; some of them are seen in Figure 6. The mechanical characteristics of a scaffold depend on the material and the process of production that influences structural parameters such as pore geometry, size, and form [204,249].

The properties of the scaffold are chosen corresponding to the application needed. To preserve their structural integrity, scaffolds must also be able to withstand the environmental stresses encountered during the operation. In addition, it is important to balance the ultimate strength and compliance with the surrounding tissue when constructing scaffolds for load-bearing applications, such as in bone TE. The scaffold should therefore have similar mechanical characteristics to the bone [250,251]. If the scaffold’s mechanical strength exceeds that of the healthy bone underlying it, stress shielding may occur, causing atrophy or tissue loss. The mechanical characteristics of a polymer scaffold depend closely on the molecular weight and the crystalline properties of the polymer that also affect the degradation rate and mechanism [252].

To facilitate quick tissue regeneration, the scaffold should have the right mechanical features and degradation rate with the bioactive surface. After implantation, it is extremely important to preserve the mechanical strength of the scaffold system for the reconstruction of rough load-bearing tissues such as bones and cartilages [253,254]. Therefore, the scaffold should be of equal mechanical strength to that of the tissue. For biodegradable scaffolds, this strength is destined to reduce overtime, but the combined strength of the newly grown tissue and the decaying scaffold should be comparable [210], since the regenerated tissue fills the degraded scaffold place. It is, therefore, necessary that one or more of the following rheological parameters is evaluated: tensile strength/compressive strength, Young’s modulus, maximum strain, and flexural modulus [255]. Table 6 illustrates a few of the biopolymer scaffold’s mechanical properties including strength, Young’s modulus, and elongation at break.

## 5. Commercial Status of Biopolymers

Currently, various biopolymers competing successfully in the global market due to their unique characteristic properties have a huge demand in biomedical applications. Besides, there is no surprise that improving human health and lifespan contribute to one of the fastest-growing markets for TE and regenerative medicine products. To help with this, the industry has been developing new biomaterial-based products, which include both synthetic and naturally derived materials [203,272,273,274]. A few commercial polymeric materials along with their trade name available for different biomedical applications are summarized in Table 7.

## 6. Scaffold Fabrication Techniques

**Figure 7:**
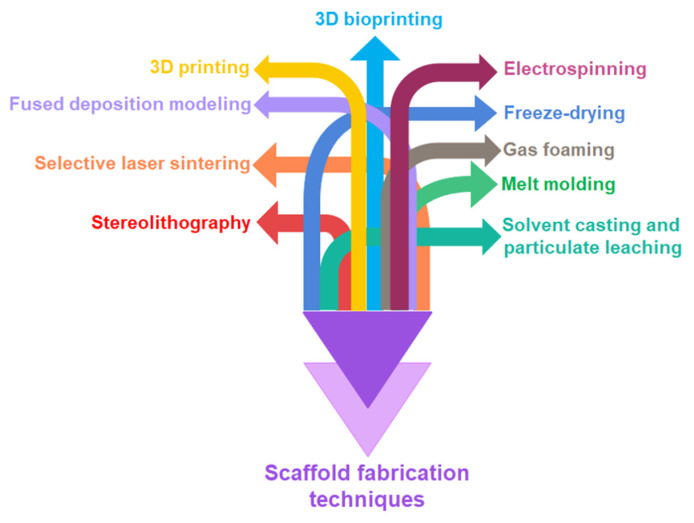
Conventional and advanced scaffold fabrication techniques.

Numerous techniques have been developed over the years for biodegradable polymers processing and fabrication of different types of scaffolds. The conventional as well as new advanced technologies are widely used for scaffold fabrication as shown in Figure 7.

Conventional techniques comprise solvent casting, particulate leaching, melt molding, gas foaming, freeze-drying, etc. Advanced techniques including electrospinning, stereolithography, selective laser sintering, fused deposition modelling, 3D printing, and 3D bioprinting can be used for fabricating natural as well as synthetic polymers and their composite scaffolds. Table 8 describes some of the basic advantages and disadvantages of these techniques along with a few possible scaffold materials [161,273,275,276,277,278]. The assessment of scaffold fabrication techniques should be performed by contemplating both potential advantages and disadvantages of each technique and the final product properties should match the needs of specific tissues to be regenerated.

## 7. Conclusions

In biomedical applications, scaffolds can be used ranging from regenerative engineering to controlled drug delivery and immunomodulation, and for this purpose, biomaterials have become an indispensable instrument as a scaffold material. The materials used for scaffold manufacturing must satisfy some criteria such as intrinsic biofunctionality and appropriate chemistry to stimulate molecular biorecognition by cells to induce proliferation, cell adhesion, and activation. The mechanical properties of scaffolds and kinetics of decomposition in selected materials must be adjusted to the TE application specifically to ensure the essential structural functions and to achieve the rate of formation of new tissues. The geometrical features like exposed surface area, pore distribution and porosity, and distribution affect the rate of cell penetration within the scaffold volume, the architecture of the ECM formed. The final effectiveness of the regenerative process plays a major role in scaffolding. Many biodegradable polymers of natural and synthetic origin have been established for use as biomaterials and careful consideration of the cellular environment and interactions needed is required to select a polymer for a given application. Despite advantages and disadvantages of individual materials, it is proposed that not one substance features all the perfect properties for a tissue replacement. Instead, a scaffold made from a composite containing both natural and synthetic biopolymers can permit tissue substitutes to be produced that satisfy all clinical requirements, including the specific size and kind of wound, the age of the patient, and the procedure of preparation available. Besides, scaffold design for tissue engineering includes several specifications. Many of these are dynamic and not yet well comprehended. In addition, being both bulk and biocompatible degraded, these scaffolds should possess sufficient mechanical properties to provide the neo-tissues with the necessary stress environment. To enable the entry of cells and nutrients, the scaffolds should also be porous and permeable and should demonstrate the required surface structure and chemistry for cell attachment.

## Figures and Tables

**Table 1:** Comprehensive analysis of naturally occurring and synthetic biopolymers along with their advantages and disadvantages.

Polymer			Structure	Desirable Properties and Advantages	Disadvantages	Ref
**Natural polymer-,**	**Polypeptide-, and Protein-based scaffolds**	**Collagen** 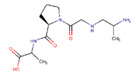	Triple helical structure held together by hydrogen bonds. Major amino acid groups include: Glycine Proline Hydroxyproline	Favorable for cell adhesion, proliferation, differentiation, and ECM secretion. Excellent biocompatibility. Biodegradability. Low toxicity. Rough surface morphology. Low immunogenicity. Weak antigenicity.	Low mechanical strength. Difficult disinfection. The deformation and contraction of collagen-based scaffolds have restricted their use in load-bearing tissues. Poor stability in an aqueous environment. Potential for antigenicity through telopeptides.	[52,124,125]
		**Silk fibroin** 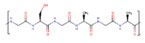	Consists of short amino acid side chains that assemble into β-sheet structures.	SFs are sturdy, lightweight, and have exceptional strength and elasticity. Osteoconductivity. Biocompatible. Deliver good support for cell adhesion and proliferation without initiating cell toxicity. Promote cell migration and vascularization. Moderately degradable. Thermostable (up to ∼250 °C). Commonly employed as a cell carrier for cell seeding on scaffolds.	Prolonged presence of silk may induce degradation, which releases certain residues or degraded products that may prompt the immune response.	[64,126,127]
		**Fibrinogen and fibrin** 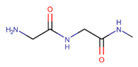	Fibrinogen: Dimer consisting of three pairs of polypeptide chains (Aα, Bβ, and γ)	Biocompatibility. High affinity for biological surfaces and molecules. Promotes cellular interactions. Variety of cell-adhesive/binding properties. Nonimmunogenicity.	Low mechanical strength. Quick rate of degradation.	[67,128,129,130]
		**Gelatin** 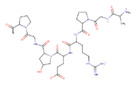	Contains glycine residues, proline, and 4-hydroxyproline residues	Better infiltration, adhesion, spreading, and proliferation of cells on resulting scaffolds. Good stability at high temperature in a broad range of pH. Biodegradability. Osteoconductivity. Non-immunogenic. Low antigenicity.	Bioactivity is questionable in higher-order gelatin structures in scaffolds. Low stability in physiological conditions.	[60,131,132]
		**Keratin**	It is a cysteine-rich fibrous protein that associates with intermediate filaments (IFs) forming the bulk of the cytoskeleton and epidermal appendageal structures	Facilitates cell adhesion and proliferation. Unique chemistry afforded by high sulfur content. Propensity for self-assembly. Intrinsic cellular recognition. Intrinsic biological activity. Cytocompatibility. Gradual degradation.	Poor mechanical properties. Quick loss of mechanical integrity.	[133,134]
	**Polysaccharide-based scaffolds**	**Starch** 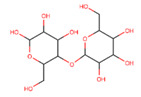	Comprised of carbohydrates. The structure consists of two types of alpha glucan which are amylose and amylopectin.	Biocompatible. Thermoplastic behavior. Non-cytotoxic. Guides various developmental stages of cells. Hydrophilicity. Good substrate for cell adhesion. Good biodegradation period.	Very high water uptake. Low mechanical strength. Unstable for long-term application. Chemical modifications may lead to toxic byproducts and reduce the rate of degradation.	[135,136]
		**Chitin/chitosan** 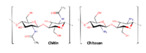	Chitin: N-acetyl glucosamine and N-glucosamine monomers Chitosan: N-deacetylated derivative of chitin	Accelerates tissue repair. Prevents formation of scar tissue. Promotes cell adhesion. Non-toxic and non-allergenic. Bioactivity. Anti-inflammatory. Osteoconductivity. Hemostatic potential. Scaffolds could be used for a longer period. Chitosan-based scaffolds can immobilize growth factors.	Poor mechanical strength and stability. High viscosity and low solubility at neutral pH. Rapid in vivo degradation rate.	[75,137,138,139,140,141,142,143,144,145,146,147,148,149]
		**Agarose** 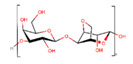	Contains repeating units of agarobiose (a disaccharide of D-galactose and 3,6-anhydro-l-galactopyranose).	Excellent biocompatibility. Thermo-reversible gelation behavior. Exceptional electroresponsiveness. Suitable medium for cell encapsulation. Non-immunogenic.	Low cell adhesion. Nondegradability due to the absence of an appropriate enzyme in the body.	[140,141,142]
		**Alginate** 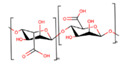	Made up of mannuronate and gluronate monomers. Different block configurations give rise to different materials properties. Mainly made up of carboxyl groups.	Mimicking function of the extracellular matrix of body tissue. Thickening/gel-forming agent. Biocompatibility. Cytocompatibility. Biodegradability. Bioabsorbable. Hydrophilicity.	Difficult to sterilize. Low cell adhesion. Poor mechanical characteristics.	[143,144,145]
		**Cellulose** 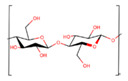	Polysaccharides are formed by many D-glucose units connected by glycosidic bonds.	Stable matrix for tissue engineering applications. Better mechanical strength. Hydrophilicity. Biocompatibility. Cytocompatibility. Bioactivity.	Cellulose in the human organism behaves as a nondegradable or very slowly degradable material.	[146,147,148]
		**Hyaluronic acid** 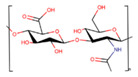	It is a linear, anionic, non-sulfated glycosaminoglycan with a structure composed of repeating disaccharides units: β-1,4-D-glucuronic acid and β-1,3-N-acetyl-D-glucosamide.	Encapsulation capability. Cell activity. HA scaffolds are frequently used in the case of both hard and soft tissue regeneration. Nonimmunogenic. Nonantigenic. Biocompatibility. Osteocompatibility.	Brittle; mechanical properties need fine-tuning via chemical modification. Low biodegradability in the crystalline phase.	[81,149,150,151]
		**Glycosaminoglycans** 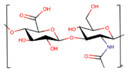	Consist of repeating disaccharides linked by glycosidic bonds creating individual complex structures.	Biocompatibility. Anticoagulant activity. Antithrombotic activity. Anti-inflammatory. Have multiple regulatory functions, e.g., in the anticoagulation of blood, inhibition of tumor growth, and metastasis. Control the inflammatory processes.	Very fast degradation. Potential risk of contamination with infectious agents.	[152,153]
**Synthetic polymers**		**Poly(ƹ-caprolactone) (PCL)** 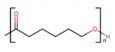	Aliphatic semicrystalline polyester.	Controls cell proliferation and angiogenesis. Slow degradation rate (lower than that of PLA and PLGA). Non-toxic. Cytocompatibility. Good mechanical properties. Degraded by hydrolysis or bulk erosion.	Low bioactivity. Hydrophobicity of PCL is another major issue that hinders wound healing application. Some problems related to withstanding mechanical loads.	[154,155,156]
		**Polylactic acid (PLA)** 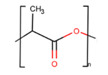	Highly crystalline.	Biocompatible. Cytocompatibility. Thermal stability. Excellent mechanical strength. Good degradation rate. Nontoxic degradation products.	PLA-based materials suffer from the lack of ideal surface chemistry that could aid cell adhesion and proliferation. Brittleness. Poor thermal stability. Hydrophobicity.	[92,157,158]
		**Polylactic-co-glycolic acid (PLGA)** 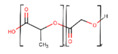	The copolymer of hydrophobic PLA and hydrophilic PGA.	Excellent cell adhesion and proliferation. Good mechanical properties. Features faster degradation than either PGA or PLA. Wide range of degradation rates.	Poor osteoconductivity. May develop biocompatibility problems.	[159]
		**Polyglycolic acid (PGA)** 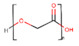	Linear highly crystalline aliphatic polyester.	Biocompatible. High tensile modulus. High melting point. Undergoes bulk degradation. Hydrophilicity.	High sensitivity to hydrolysis. Difficult to obtain porous PGA scaffolds without toxic solvents.	[160,161]
		**Polyhydroxybutyrate (PHB)** 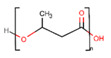	It is a homopolymer having a stereoregular structure with high crystallinity. Naturally occurring b-hydroxy acid.	Non-toxic. Biostable. Biocompatible. Advantages over PLA and PGA. Slow rate of degradation. Can be obtained naturally.	Inherent brittleness and rigidity. Thermal instability during melt processing impedes its commercial application.	[159,162,163]
		**Polypropylene fumarate (PPF)** 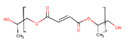	Linear and unsaturated copolyester based on fumaric acid.	Biocompatibility. Crosslinked PPF matrices have high mechanical strength. PPF degrades in the presence of water into propylene glycol and fumaric acid, the degradation products that are easily cleared from the human body by normal metabolic processes. Non-toxic.	It is a viscous liquid at room temperature (21 °C), making the handling of the polymer somewhat cumbersome	[159,164,165]
		**Poly(ethylene glycol) (PEG)** 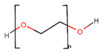	Synthesized using ring-opening polymerization of ethylene oxide.	Non-ionic. Biocompatible. Elasticity. Bioadhesive. Mucoadhesive. Hinders protein adsorption. Hydrophilic. PEG as a blank template can be modified to different moieties to pass different requirements of a skin substitute like cell adhesion, short-term degradation, and minimum inflammation. Non-immunogenic.	Lacks cell-interactive character due to its bio-inert nature. Nonreactive, creates insoluble networks.	[123,166,167]
		**Polyurethane (PU)** 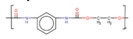	Urethane groups are the major repeating units. Synthesized by reactions of di- or polyisocyanates (hard segments) with di- or polyols (soft segments) via the catalyzed polymerization process.	Bio- and hemocompatibility. Nontoxic. Biodegradable. Non-allergenic. Non-sensitizing. Excellent mechanical properties. High flexural endurance and fatigue resistance.	PUs are less compatible with blood and found unsuitable for in vivo drug delivery application. Limited stability in vivo.	[113,168]
		**Polyvinyl alcohol (PVA)** 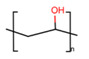	Semicrystalline polyhydroxy polymer. Prepared via hydrolysis of poly(vinyl acetate).	Biocompatible. Nontoxic. Noncarcinogenic. Displays a reduced protein-binding tendency, relatively higher elasticity and water content; a highly hydrated water-soluble synthetic polymer. Has relatively similar tensile strength to human articular cartilages. Good lubrication.	Lack of cell-adhesive property. Less ingrowth of bone cells.	[169,170,171]
		**Polypropylene carbonate (PPC)** 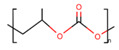	Product of alternating copolymerization of propylene oxide and CO_2_. Amorphous.	Biodegradable amorphous polymer because of the aliphatic polycarbonate ester structure on its backbone. No inflammatory response. Thermoplastic behavior. Biocompatibility. Impact resistance.	PPC has shortcomings such as viscous flow at room temperature and a relatively large brittleness at low temperature. Poor thermal and processing properties. Cell attachment to PPC is very limited due to its highly hydrophobic nature.	[172,173,174]

**Table 2:** Comprehensive analysis of natural biopolymer blends (composites) along with their fabrication route, properties, biological assessment, and characteristics.

Natural–Natural Biopolymer Composite Scaffold Material	Fabrication Method	Properties Considered	Biological Assessment	Characteristics	Scaffold Application	Ref.
Collagen	Freeze-drying	Porosity: 98.8%. Young’s modulus: ~240 KPa (after 8 weeks).	Cell seeding efficiency: 93.8 ± 2.0%. In vivo implantation. Histological and immunohistochemical evaluations.	The highest stimulating effect was seen on gene expression and cartilaginous matrix protein production and also on cartilage regeneration. The findings of vivo implantation showed that the pore size had no apparent effect on the proliferation of cells.	Cartilage regeneration	[176]
Collagen/gelatin/chitosan (40–20–40%)	Freeze-drying	Porosity: 61.34% ± 2.53%. Density: 0.0522 g/cm^3^. Swelling: 34.8% (in PBS). Stress: 4 MPa.	ABTS (2,2′-azino-bis(3-ethylbenzothiazoline-6-sulfonic acid)) % of inhibition = 3.0268. Maximum zone of inhibition: 12 mm (*Escherichia coli*) and 24 mm (*Staphylococcus aureus*).	Wound-healing properties. Obviates the need to remove the material later or leave materials in the body. The efficiency of the antimicrobial activity decreases over time.	Tissue engineering	[177]
Collagen–chitosan (7:3)	Lyophilization	Swelling: ~8%. In vitro degradation: > 15% (7 days in PBS containing a lysozyme enzyme).	Protein adsorption: 0.65 (optical density (OD562nm)).	Higher protein absorption. Decrement in the rate of degradation compared to a pristine polymer.	Tissue regeneration	[178]
Cellulose–collagen (5:1)	Freeze-drying	Water uptake: 400%. Contraction: ~3%.	Cell proliferation: 9 × 10^4^ (number of cells in three days). Percentage of neovessel-occupied area: ~4%. Percentage of blood vessel-occupied area (number of cells/mm^2^): 4.06 ± 0.8%.	Excellent physical stability. Provides 3D environment for good cell retention and proliferation. Provides microenvironment for induction of osteogenic differentiation of mesenchymal stem cells extracted from umbilical cord blood (UCB-MSCs). Collagen’s low mechanical properties are a weak point.	Bone tissue engineering	[179]
Silk fibrils/chitosan (3:4)	Freeze-drying	Tensile strength: 40.1 ± 1.9 MPa. Compressive modulus and strength at 60% strain in dry state: 81.7 ± 6.2 kPa and 78.5 ± 3.6 kPa.	/	Improved thermal stability and mechanical strength. Due to the addition of the silk nanofibrils SNF, the maximum thermal decomposition temperature is increased. The ECM composition is imitated by SNF/CS nanocomposites and thus offers choices for the creation of novel biomaterials.	Would dressing, tissue engineering scaffolds, flexible biodevices	[180]
Chitosan/SF (7:3)	Lyophilization	Elastic modulus: 5.3 ± 0.2 MPa. Tensile strength: 3.1 ± 0.7 MPa. Elongation at break: 56 ± 7.4%. Suture retention strength: 1.96 ± 0.25 N. Swelling index: 348 ± 39%.	Cell isolation and culture. Cell adhesion and proliferation. Immunohisto-chemistry.	Mechanical strength, oxygen, and nutrient permeability prevent fibrous scar tissue invasion.	Nerve regeneration, cartilage regeneration	[181]
SF (7 w/v%)/chitosan–gelatin (1:2) cross-linked with methanol and glutaraldehyde	Freeze-drying	Pore size: 175 ± 15 µm. Porosity: 78%. Tensile strength: 11 ± 0.26 KPa. Young’s modulus: 40 ± 3.8 KPa. Breaking strain: 27.5 ± 2.02%. Contact angle (°): 58 ± 7. Swelling index: ~90%. Degradation: ~55% (four weeks).	Absorbance (490 nm): 1.1 (six days). Histological assessment.	Mechanical features similar to those of the native soft tissues were seen in the formed scaffolds. High degradation rate. The mechanical strength and degradation rate improved by the addition of silk fibroin to the composites. Compared with silk fibroin alone, the composite scaffolds have increased endothelial cell attachment and growth.	Tissue engineering	[182]
Oxidized alginate/gelatin/SF (13:17:10 w/v%)	Electrospinning	Pore size: 412.58 ± 86.2 µm. Porosity: 80.9 ± 3.1%. Water uptake: > 100%. Degradation: ~50% (four weeks). Young’s modulus: 1.84 MPa.	Cell viability and proliferation: ~0.7 for seven days (OD, 562 nm).	Non-toxic and supports AMSC (adipose-derived mesenchymal stem cells) proliferation. Higher thermal stability.	Regenerative medicine, skin tissue engineering	[183]
Collagen–HA (15 wt.%)	Freeze-drying	Relative density: 0.0121 ± 0.0008. Porosity: ~85%. Degradation rate: 13.3% (seven days). Young’s modulus: 6.73 ± 0.41 KPa. Collapse plateau modulus: 3.17 ± 0.36 KPa. Elastic collapse stress: 625 ± 29 Pa. Elastic collapse strain: 0.10 ± 0.01.	Cell culture. Immunohisto-chemistry.	Collagen–HA scaffolds that favor the differentiation of neural stem cells into neuronal cells in vitro in tandem with some mechanical behaviour of brain tissue.	Brain tissue engineering	[184]
Alginate/cellulose nanocrystals–chitosan–gelatin	Layer-by-layer assembly and then freeze-drying	Porosity: 77.4%. Compressive strength: ~0.28 MPa. Degradation rate: ~23% (two weeks).	Cell proliferation: 3.8 for five days (OD, 562 nm). Relative ALP (alkaline phosphatase) activity: 1.5 after six days of incubation.	A strong 3D architecture with a well-defined porous structure improves compressive strength and controlled biodegradation.	Bone tissue engineering	[185]

**Table 3:** Comprehensive analysis of natural–synthetic biopolymer blends (composites) along with their fabrication route, properties, biological assessment, and characteristics.

Natural–Synthetic Biopolymer Composite Scaffold Material	Fabrication Method	Properties Considered	Biological Assessment	Characteristics	Scaffold Application	Ref.
PCL/collagen	Electrospinning	Tensile strength: 0.9 MPa (explanted in one month). Graft patency and geometry, structural integrity.	Cell culture, histology, cell adherence, and resistance to platelet adherence.	Maintains a high degree of patency and structural integrity in vivo without eliciting abnormal inflammatory response over one month. Capable of promoting endothelial and muscle cell growth under conditions of pulsatile flow. Issues such as immune response, scaffold cell remodeling, and in vivo development of thrombosis have not been described.	Vascular tissue engineering	[187]
Chitosan/PLLA/pectin (50:25:25)	Freeze drying	Avg. pore size: 49–164 μm. Porosity: 81 ± 1.97%. Swelling ratio: 1.6 (36 h). Degradation: ~38% (28 days).	Cell proliferation: 0.7 (seven days). Hemocompatibility: 1.97% hemolysis. Biopsy collection and chondrocytes culture. Cytocompatibility assay. Cell viability analysis. Histopathological. Immunofluorescence studies.	Displays an increase in compressive strength, controlled swelling property, lower degradation behavior, and hemocompatibility according to the polymeric proportion. The in vivo study accompanied by histological analysis demonstrated the neo-cartilage tissue regeneration potential of the cell–scaffold construct.	Neo-cartilage tissue regeneration, surgical manipulation	[188]
PLA/chitosan	Fused filament fabrication (3D printing)	Tensile strength: 44.56 MPa. Compression strength: 47.15 MPa. Flexural strength: 156.96 MPa.	/	The established scaffold has a considerably higher flexural strength than compression strength and tensile strength, which makes the scaffold ideal for dynamic movements. Lower tensile strength and compression strength. PLA/chitosan scaffolds have a lower strength than PLA scaffolds.	Clinical purposes	[189]
Alginate-coated PLLA/PLGA (95:5, w/w)	Lyophilization	Pore size: 39 ± 24 μm. Porosity: 60–65%. Compressive modulus: 1415 ± 153 kPa. Compressive strength: 128 ± 18 kPa. Degradation: 40% (eight weeks).	Cell proliferation: ~25 × 10^4^ (number of cells in 15 days). Cell morphology.	Cell proliferation rate is low on alginate-coated scaffolds. Cells are also shown to become more branched in the presence of alginate.	Designing engineered tissues	[190]
PLLA/gelatin (6%)/osteo (1.5%)	Electrospinning and 3D printing (FDM: Fused deposition modeling)	Tensile strength: 17.7 ± 1.8 MPa. Porosity: 44.1%.	Bioactivity. Cell culture. Cytotoxicity. Proliferation.	The presence of gelatin and an osteogenic drug on the surface of 3D-printed PLLA scaffolds offers mineralization of the samples proving its bioactivity.	Nasal cartilages and subchondral bone reconstruction	[191]
PLLA/PCL/HA	Electrospinning associated with electrospray	Thickness: 16 ± 4 µm. Young’s modulus: 2.99 ± 0.63 MPa. Tensile strength: 11.32 ± 1.94 MPa. Elongation at break: 131.83 ± 6.82 %.	Metabolic activity of MC3T3-E1 cells/area: ~1500 (RFU/mm^2^) (where RFU: relative fluorescence units). Total number of colony-forming units (CFU) per mL of *Staphylococcus aureus* adhesion: 17 × 10^4^.	Enhancement of mechanical strength. The adhesion and proliferation of osteoblast cells and the fiber alignment are induced to increase the metabolic activity of the cells.	Tissue engineering	[192]
CS/PVA/ methylcellulose	Combination of film casting and lyophilization methods	Porosity: 88%. Young’s modulus: 119.3 ± 0.4 MPa. Tensile strength: 8.40 ± 0.3 MPa. Elongation at break: 8 ± 0.9 %. Degradation: 39 ± 2.0%. Swelling degree: 71 ± 3.6%.	Bacteriostatic rate: 81.2 ± 3.9 % (*E. coli*), 79.3 ± 4.1% (*S. aureus*). Cell proliferation assay of L929 cells: ~1.6 (seven days).	The compatibility between CS and PVA has improved by adding MC. Along with the high swelling rate, the mechanical characteristics of these scaffolds are greatly improved. The biocompatibility test showed that there is no cytotoxicity in the various MC scaffolds.	Drug delivery vehicles and skin tissue engineering	[193]
PCL/PPy	Electrospinning (ES)	Young’s modulus:10.50 MPa. Tensile strength:15.26 MPa. Strain at break: 320.07%. Contact angle: 93.40 ± 0.36. Conductivity: 15.60 × 10^−7^ (S/m).	Cell viability with ES: 1.95 (OD, 450 nm) (seven days). ALP activity with ES: 8.5 (mM) (14 days). ARS (Alizarin red S) staining with ES: 2.35 (21 days).	In electric stimulation conditions, PCL/PPy show improved MC3T3-E1 cellular adhesion, proliferation, and differentiation. Increased simulated body fluid (SBF)-biomineralization has been shown for PCL/PPy conductive scaffolds.	Bone tissue engineering	[194]
Chitosan(CS)/PCL(P)/gelatin(G)	Electrospinning followed by freeze-drying	Pore size: 8.8 ± 1.4 μm. Porosity: 47%. Swelling ratio: 1270 ± 16%. Contact angle: 46.9 ± 2.0°. Maximum stress: 0.372 ± 0.029 MPa. Strain at failure: 80%. Young’s modulus: 0.4 MPa. Degradation rate: 20% (three months).	Cell biocompatibility analysis. Cell viability analysis. Collagen secretion measurement. Cell attachment analysis. Hemostatic effect in vitro. Biodegradability analysis in vivo. Cell infiltration analysis.	The composite scaffolds had good blood coagulation abilities because of the hemostatic properties of CS and the porous structure. Filaments and tiny pores in composite CS–PG scaffolds may serve as effective barriers and prevent cell infiltration.	Periodontal regeneration	[195]
PCL/PVP (polyvinylpyrrolidone)	E-jet 3D printing	Jetting morphology. Printed structures features.	Cell viability: 95 ± 3.5% (five days). Normalized cell density: 500 (five days).	The composite PCL/PVP scaffolds are printed with the controllable diameter of the filament (~10 μm) that is close to living cells. Experiments in cell culture found that printed scaffolds have excellent biocompatibility and support in vitro cell proliferation.	Cartilage regeneration	[196]
PLA/regenerated cellulose (RC)	Electrospinning and freeze-drying techniques	Porosity: 96.3 ± 0.2%. Density: 32.4 ± 0.2 mg/cm^3^. Water absorption capacity: 3500%. Youngs modulus: 54.9 kPa. Compressive stress at 80% strain: 120 KPa. Degradation: 14.66% (56 days).	In vitro biomineralization.	Increased hydrophilicity and biological activity. The properties of high water absorption, hierarchical cellular structure, and rapid recovery from 80 percent strain are presented by PLA/RC nanofiber-reconfigured scaffolds.	Bone tissue engineering	[197]
PLA/cellulose nanocrystals	Electrospinning	Modulus: 1.32 MPa. Toughness: 2.07 mJ/m^3^.	Cell viability: ~240 % (five days). Mineralization (A562): 0.3 (14 days). Cell morphology. Real-time PCR analysis. In vivo study and histological analysis of bone regeneration.	Outstanding adhesion and mineralisation. Enhanced osteogenesis by manufacturing electrospun scaffolds. Improved bone regeneration in a scaffold-treated group.	Bone tissue engineering	[198]
PCL/polyaniline (0.1 wt.%)	Screw-assisted extrusion-based 3D printing	Pore size: 305.9 ± 35.5 μm. Porosity: 48.16 ± 1.071%. Contact angle: 83°. Compressive Young’s modulus: 68.35 ± 5.15 MPa. Compressive strength: 6.45 ± 0.16 MPa. Conductivity: 2.46 ± 0.65 × 10^−4^ S/cm.	Cell viability: 88% (one day).	The highest cell viability with cytocompatibility in cell culture has been demonstrated for up to 21 days.	Bone tissue engineering	[199]
PBS/cellulose nanocrystals (5 wt.%)	Two-step depressurization in a supercritical carbon dioxide (Sc-CO_2_) foaming process	Compressive strength: 2.76 MPa. Contact angle: 71.7°. Porosity: 95.2%. Degradation rate: 20.5% (six weeks).	Cell viability (% of a living cell): 98.05 (seven days). Cell proliferation (OD values): ~1.0 (seven days).	The strong in vitro biocompatibility has been demonstrated and can provide effective cell attachment and proliferation environment.	Tissue engineering	[200]

**Table 4:** Degradation mechanism of biodegradable polymer scaffolds.

Scaffold Material	Degradation Mechanism	Degradation Duration (Weeks)	Degradation Rate (%)	Solvent	Application	Ref.
Alginate	Enzymatic	4	>70	DMEM + FBS	Bone and cartilage tissue substitutes	[217]
Gelatin	Hydrolysis, dissolving, transformation, and enzyme-catalyzed decomposition	2.5	94.9	Lysozyme	Cartilage cells	[218]
Chitosan/gelatin	Enzymatic	4	28 ± 3.5	PBS	Tissue engineering	[219]
Chitosan	Enzymatic	4	∼60	Lysozyme	Cartilage regeneration	[220]
Silk fibroin/chitosan			50			
Silk fibroin/hyaluronic acid	Enzymatic	3	∼47	Collagenase IA solution	Soft tissue engineering	[221]
Silk fibroin			∼72			
Chitosan/gelatin	Enzymatic	3	50–60	PBS with lysozyme	Biomedical applications	[222,223]
Collagen	Enzymatic	2	71	PBS	Tissue engineering	[224]
Collagen/PLLA	Hydrolysis and enzymatic		5			
Starch/PVA	Hydrolytic	4	27.1	Simulated body fluid (SBF)	Bone tissue engineering	[225]
Chitosan/PVP–PLGA	Hydrolytic	4–6	100	PBS	Allergic rhinitis and chronic sinusitis	[226]
PLA	Enzymatic	32	80	Simulated body fluid (SBF)	Tissue engineering	[227]
PGA	Hydrolytic	1–6	50	PBS	Tissue-engineered vascular grafts	[228]
PCL	Hydrolytic (surface erosion)	24	7	PBS	Drug delivery and tissue engineering	[229]
PLGA	Hydrolytic	6	~50	PBS	Tissue engineering, drug carriers, and sensors	[230]
PGA		3	60			
PCL/PLLA	Hydrolytic	5	14	NaOH solution	Bone tissue engineering	[231]
Polyurethane copolymers	Hydrolytic	8	∼10	PBS	Soft tissue engineering	[232]
PLA/thermoplastic polyurethane	Hydrolytic	4	∼10	PBS	Medical and tissue engineering	[233]

**Table 5:** The porosity and pore size of polymer scaffolds.

Scaffold Material	Fabrication Method	Pore Size (μm)	Porosity (%)	Application	Ref.
Trabecular bone	NA	/	50–90	NA	[240]
Cortical bone		/	3–12		
Collagen	Freeze-drying	150–250	98.8 ± 0.1	Cartilage regeneration	[177]
Collagen	Freeze-drying	/	96.05 ± 0.11	Bone tissue engineering	[241]
Gelatin	Freeze-drying	∼50–100	~98	Cartilage cells	[218]
Collagen/chitosan	Freeze-drying	2–5	41.5% ± 2.69	Tissue engineering	[178]
Gelatin/chitosan		5–10	81.02% ± 1.04		
Collagen/gelatin/chitosan		10–20	61.34% ± 2.53		
Silk fibroin	Freeze-drying	70 ± 23	92	Tissue engineering	[183]
Chitosan/gelatin		280 ± 31	67		
Silk fibroin/chitosan/gelatin		153 ± 18	80		
PCL	Electrospinning	~44–64	~90	ECM for tissue engineering	[242]
PCL	Fused deposition modelling	/	70	Bone regeneration	[229]
PCL	Extrusion	/	49.0 ± 1.4	Biomedical applications	[28]
PCL/cellulose nanofibers		/	49.5 ± 2.1		
Alginate	Freeze-drying	250–320	85 ± 3.1	Bone and cartilage tissue engineering	[217]
Alginate dialdehyde–gelatin (ADA–GEL)	Freeze-drying	~200	~90	Bone tissue engineering	[243]
PLA	Melt blending and hot pressing	80.01	79.88	Tissue engineering	[227]
PPC	Gas foaming–salt leaching method	418 ± 84	92.4	Tissue engineering	[244]
PGA	Electrospinning	157.9 ± 30.5	91.5 ± 4.1	Tissue-engineered intestines (TEI)	[245]
PCL		45.0 ± 12.6	67.9 ± 2.9		
PGA/PLLA		84.7 ± 23.2	81.9 ± 3.3		
CollaTape		54.4 ± 10.6	86.7 ± 3.4		
CollaTape/PLLA		45.2 ± 22.5	76.6 ± 3.9		
Collagen/PLLA	Lyophilizing	150–250	>95	Tissue engineering	[224]

**Table 6:** Mechanical properties of scaffold materials.

Scaffold Material	Scaffold Fabrication Method	Young’s Modulus (MPa)	Strength (MPa)	Elongation (%) at Break	Scaffold Application	Ref.
Cortical bone	NA	15–20 × 10^3^	100–230	/	NA	[35,256]
Trabecular bone		0.1–2 × 10^3^	2–12	/		
Cancellous bone		20–500	4–12	/		
Cartilage		0.7–15.3	3.7–10.5	/		
Tendon		0.143–2.31 × 10^3^	24–112	/		
Silk fibroin (SF)	Solvent casting	310 ± 90	22.8 ± 13.7	1.3 ± 0.3	Soft tissue engineering	[257]
Gelatin (G)		370 ± 80	95.3 ± 25.6	5.3 ± 1.4		
SF/G (50/50)		460 ± 70	89.4 ± 12.9	3.2 ± 0.6		
Collagen	Solution casting	/	57 ± 6	16.3 ± 1.3	Biomedical applications	[258]
Collagen/cellulose nanofibers (8%)			156 ± 5	23.06 ± 1.3		
Alginate	Freeze-drying	65 ± 13 ×10^−3^	326 ± 49 × 10^−3^	/	Bone tissue engineering	[243]
Alginate–gelatin–bioglass (5 w/v%)		417 ± 33 × 10^−3^	908 ± 117 ×10^−3^			
Silk fibroin	Freeze-drying	70 ± 1.01 × 10^−3^	14 ± 2 × 10^−3^	27.5 ± 6.2	Tissue engineering	[183]
Chitosan/gelatin		20 ± 1.3 × 10^−3^	5.6 ± 0.2 × 10^−3^	37.9 ± 3.8		
Silk fibroin/chitosan/gelatin		27 ± 1.4 × 10^−3^	7.4 ± 0.3 × 10^−3^	36.6 ± 3.5		
Chitosan	Lyophilization	6.8 ± 0.5	4.7 ± 0.4	62 ± 8.7	Nerve regeneration, cartilage regeneration	[259]
Chitosan/silk fibroin (7:3)		5.3 ± 0.2	3.1 ± 0.7	56 ± 7.4		
Chitosan/silk fibroin (5:5)		3.4 ± 0.3	2.1 ± 0.5	33 ± 4.8		
PCL	Cryogenic plotting/melt plotting	17.00 ± 0.75	1.71 ± 0.37	/	Hard tissue regeneration	[260]
Collagen		0.55 ± 0.03	0.024 ± 0.003			
Core (PCL)–shell (collagen/alginate) (shell–core = 0.18)		8.68 ± 1.14	1.28 ± 0.17			
PCL/gelatin/hyaluronic acid fibers	Electrospinning	/	7.9 ± 0.8	69	Glioblastoma extracellular matrix	[261]
Gelatin	Electrospinning	105	2.50	64	Tissue engineering	[262]
PCL		4.98	2.70	126		
Gelatin/PCL		30.8	1.29	138		
PLLA	Electrospinning	55.93 ± 2.11	3.05 ± 0.21	37.3 ± 3.9	Tissue engineering	[263]
PLLA/PCL (90/10)		18.11 ± 0.94	2.75 ± 0.09	66.5 ± 8.6		
PLLA/PCL (50/50)		6.21 ± 0.64	1.58 ± 0.16	94.6 ± 7.5		
PGA	Melt compounding or lamination	7000	115	16.4	Biomedical applications	[264]
PHB	Solution-cast	3500	40	5	Therapeutic applications	[265]
Polypropylene	/	1700	38	400	Therapeutic applications	[266,267]
Low-density polyethylene	/	200	10	620	Therapeutic applications	[265]
Polystyrene	/	3100	50	/	Biomedical applications	[267]
PVC	/	300–2400	10–60	12–32	Biomedical applications	[266]
PLA	/	2400	53	5	Biomedical applications	[268]
PCL	Electrospinning	7.5 ± 0.7	1.5 ± 0.7	417 ± 58	Vascular cells	[269]
PCL/collagen (dry)		3.8 ± 5.6	8.3 ± 1.2	62 ± 5		
PCL/collagen (wet)		2.7 ± 1.2	4.0 ± 0.4	140 ± 13		
PLGA	/	2000–4000	40–90	< 10	Biomedical applications	[270,271]
PET	/	3500	47	2–83	Biomedical applications	[264]
PA 6	Melt compounding followed by injection moulding	1947 ± 164	56 ± 1.0	70	Biomedical applications	[182]

**Table 7:** Commercial biopolymers for different biomedical applications.

Polymer	Biomedical Application	Trade Name
Collagen	Provides an increased surface area for cell attachment, growth, and migration for tissue engineering applications	SpongeCol^®^
	SphereCol^®^ provides a 3D bio-scaffold which is optimal in many cell culture procedures	SphereCol^®^
	VitroCol^®^ is especially ideal for human cell culture systems as a coating for surfaces, for providing preparations of thin layers of cultured cells, or for use as a solid gel.	VitroCol^®^
	Skin replacement product	TransCyte^®^
Gelatin	A medical device intended for application to bleeding surfaces as a hemostatic	Gelfoam^®^
Silk	Therapeutic clothing	DermaSilk^®^
Chitosan	Natural wound care for animals—big and small	ChitoClear^®^
	Natural healing and scar recovery	ChitoCare^®^
Hyaluronic acid	Cell culture scaffolds	HyStem™
PGA	Mainly applied for absorbable sutures and also for stents, adhesion barriers, absorbable reinforcement for artificial dura, and scaffolds	BioDegmer^®^ PGA
	The first biodegradable synthetic suture (1969)	DEXON
	Bone internal fixation devices	Biofix^®^
	Medical device applications	PURASORB^®^ PG
	Absorbable mesh for temporary wound and organ support	Safil^®^ Mesh
PLA	Meniscus repair fixation devices	The Meniscus Arrow (Bionx Implants, Inc., Blue Bell, PA), Clearfix Screw (Mitek, Norwood, MA)
	Fixed installations such as bone plates, bone screws, surgical sutures, spinning	Revode 100 series Revode 200 series
PGLA (Poly(glycolide-co-L-lactide))	Mainly applied for absorbable sutures and also for stents, scaffolds, adhesion barriers, artificial dura, and guided tissue regeneration (GTR) membranes	BioDegmer^®^ PGLA
	A temporary wound or organ support	VICRYL™ (polyglactin 910) Woven Mesh
PGDLLA (Poly(glycolide-co-DL-lactide))	Mainly applied for GTR membranes (porous membranes) for regeneration and adhesion of lost periodontal supporting tissues caused by periodontal disease	BioDegmer^®^ PGDLLA
PLLA	Mainly applied for absorbable bone fixture and utilized for stents, scaffolds, and adhesion barriers	BioDegmer^®^ PLLA
	Orthopedic fixation devices	Bio-Anchor^®^
	Medical device applications	PURASORB^®^ PL grades
	Fabrication of medical research devices and tissue engineering research solutions, such as orthopedic or soft tissue fixation devices.	Resomer^®^ series L 206 S L 207 S L 209 S L 210 S
PDLA	Bone fixture material	BioDegmer^®^ PDLA
	Medical device applications	PURASORB^®^ PD grades
PDLLA	Mainly applied for the coating of suture	BioDegmer^®^ PDLLA
	Medical device applications	PURASORB^®^ PDL grades
	Form scaffolds make it a useful biomaterial in biomedical and tissue engineering	Resomer^®^ R 207 S
PCL	Medical device applications	PURASORB^®^ PC
67% PGA: 33% trimethylene carbonate (TMC)	Soft tissue reinforcement	BIO-A^®^

**Table 8:** Biopolymer scaffold fabrication techniques.

Fabrication Method	Advantages	Disadvantages	Materials	Ref.
**Solvent casting and particulate leaching:** Polymer solution poured into the mold along with an appropriate porogen. A porous scaffold is obtained at high pressure and after evaporation of organic solvents	Control over porosity, pore size, and crystallinity. Highly porous materials with interconnected pores. Simple and reproducible technique.	Limited mechanical properties, residual solvents, and porogen material. Longer processing time. This technique is mainly applied to produce thin membranes.	Different classes of synthetic polymers (e.g., PLLA, PLGA, or PEG) and natural polymers	[279,280]
**Melt molding:** Both polymers and a suitable porogen are melted together, then by cooling the polymer mixture the scaffold is obtained. In this process, the porosity is attained by dissolving the porogen in water	Independent control over porosity, pore size, pore interconnectivity, and geometry.	The requirement of high temperature for the non-amorphous polymer. Requires a residual porogen. Longer processing time. Limited mechanical properties. Expensive technique.	PLA, PGA, PLGA–gelatin, PA	[281]
**Gas foaming:** Polymer gel paste along with sieved effervescent salt particles poured into a mold and immersed into hot water. Formation of the porous matrix after the evolution of ammonia and carbon dioxide gas from salt particles of the solidifying polymer matrix	Free of harsh organic solvents. Control over porosity and pore size. Minimum loss of bioactive molecules. No need for the leaching process. High porosity > 90%.	Limited mechanical properties, inadequate pore interconnectivity. Longer processing time.	PLA, PLLA, or PLGA	[282,283]
**Freeze-drying:** A polymer solution is poured into a suitable mold and solvents are removed using a lyophiliser. This technique is mainly based on the sublimation process	High temperature and a separate leaching step not required. Highly porous materials, with random or oriented pores.	Pore size is relatively small and porosity is often irregular. Long processing time. Expensive technique.	Natural polymers like alginate, agarose, gelatin, chitosan, etc., and PGA, PLLA, PLGA, PLGA/PPF blends	[284,285]
**Electrospinning:** The electrospinning process draws a continuous narrow stream of material from a reservoir of polymer melt or solution to a collecting plate, where the material accumulates, producing the fibrous mat. This is accomplished by inducing charge buildup on the surface of the solution through the application of strong voltages	Control over porosity, pore size, and fiber diameter. High surface area. Cheap and simple.	Limited mechanical properties, pore size decreases with fiber thickness. Not applicable for all polymers. Not sufficient for cell seeding. Not sufficient for cell infiltration.	Synthetic polymers (PEO, PLGA, PLLA, PCL, PVA) and natural polymers (collagen, silk fibroin, elastin, fibrinogen, chitosan) and their composites	[286,287]
**Stereolithography (SLA):** In SLA, an object is created by selectively curing a polymer resin layer-by-layer using an ultraviolet (UV) laser beam	Creates 3D scaffolds for tissue engineering with complex geometries. Pores of multiple sizes, which can ensure a selective transport of cells versus smaller molecules.	The time required for fabrication increases cubically as resolution increases.	PPF, PEO, PEG	[288,289,290]
**Selective laser sintering:** This method selectively sinters thin layers of polymer-based mixtures in the powder form, creating solid 3D composite objects with macro-and microscale features	Highly capable of producing objects with intricate structures and shapes containing channels, overhanging features, and gradient structures. TE scaffolds with controlled porosity and customized architecture.	Incapability to use polymers in the hydrogel form. Impossibility to encapsulate cells in scaffolds. Limitation in forming sharp corners and clear boundaries, making it impossible to create small details.	Nondegradable or degradable biopolymers (e.g., PE, PCL, PLLA, PLGA, etc.), and composites can be processed into scaffolds for TE	[290,291,292]
**Fused deposition modeling (FDM):** FDM uses a layer-by-layer deposition technique, in which molten polymers or ceramics are extruded through a nozzle with a small orifice and merge with the material on the previous layer	3D models of custom-made implants cast for individual patients. FDM processes can achieve pore sizes ranging from 160 to 700 microns, with porosities ranging from 48% to 77%.	Pore anisotropy and the geometry of pore connectivity are substantially limited due to the continuous deposition process. FDM is typically limited to synthetic thermoplastic polymers, thereby eliminating many natural biomaterials and thermoset synthetic polymers.	Biodegradable materials used for this method include PCL, PLGA, polycarbonate, polypropylene, and various polyesters	[290,293,294]
**3D printing:** It is a process of reconstruction of a 3D physical model by the successive addition of material layers resulting in a 3D solid object based on CAD model design	Able to create almost any shape or geometric feature, allows defined internal architectures for implants.	The addition of a chemical binder. Post-fabrication efforts to remove the residual solvent such as vacuum drying are not completely effective; therefore, the issue of cytotoxicity in 3D printing (3DP)-fabricated scaffolds remains.	PEO, PCL, and PLGA	[290,295,296]
**3D bioprinting:** It is the 3D printing process of generating layer-by-layer 3D tissue-like structures using viable cells, an encapsulation biomaterial, and growth and differentiation factors to create a bio-printed pre-tissue that is further transferred to an incubator where it matures into a tissue	Biomimicry. Autonomous self-assembly. Small tissue building blocks.	The development of biomaterials for 3D bioprinting is still in its early stages.	Common biomaterials include natural and/or synthetic polymers and decellularized ECM	[297,298,299,300]
